# Mitochondrial MPTP: A Novel Target of Ethnomedicine for Stroke Treatment by Apoptosis Inhibition

**DOI:** 10.3389/fphar.2020.00352

**Published:** 2020-03-25

**Authors:** Yangxin Li, Jiayi Sun, Ruixia Wu, Jinrong Bai, Ya Hou, Yong Zeng, Yi Zhang, Xiaobo Wang, Zhang Wang, Xianli Meng

**Affiliations:** ^1^ Ethnic Medicine Academic Heritage Innovation Research Center, Chengdu University of Traditional Chinese Medicine, Chengdu, China; ^2^ Innovative Institute of Chinese Medicine and Pharmacy, Chengdu University of Traditional Chinese Medicine, Chengdu, China; ^3^ School of Ethnic Medicine, Chengdu University of Traditional Chinese Medicine, Chengdu, China

**Keywords:** ischemic stroke, mammalian mitochondrial permeability transition pore, mitochondrial apoptosis, ethnic medicine, prescription, monomer composition

## Abstract

Mammalian mitochondrial permeability transition pore (MPTP), across the inner and outer membranes of mitochondria, is a nonspecific channel for signal transduction or material transfer between mitochondrial matrix and cytoplasm such as maintenance of Ca^2+^ homeostasis, regulation of oxidative stress signals, and protein translocation evoked by some of stimuli. Continuous MPTP opening has been proved to stimulate neuronal apoptosis in ischemic stroke. Meanwhile, inhibition of MPTP overopening-induced apoptosis has shown excellent efficacy in the treatment of ischemic stroke. Among of which, the potential molecular mechanisms of drug therapy for stroke has also been gradually revealed by researchers. The characteristics of multi-components or multi-targets for ethnic drugs also provide the possibility to treat stroke from the perspective of mitochondrial MPTP. The advantages mentioned above make it necessary for us to explore and clarify the new perspective of ethnic medicine in treating stroke and to determine the specific molecular mechanisms through advanced technologies as much as possible. In this review, we attempt to uncover the relationship between abnormal MPTP opening and neuronal apoptosis in ischemic stroke. We further summarized currently authorized drugs, ethnic medicine prescriptions, herbs, and identified monomer compounds for inhibition of MPTP overopening-induced ischemic neuron apoptosis. Finally, we strive to provide a new perspective and enlightenment for ethnic medicine in the prevention and treatment of stroke by inhibition of MPTP overopening-induced neuronal apoptosis.

## Introduction

**Figure 1:**
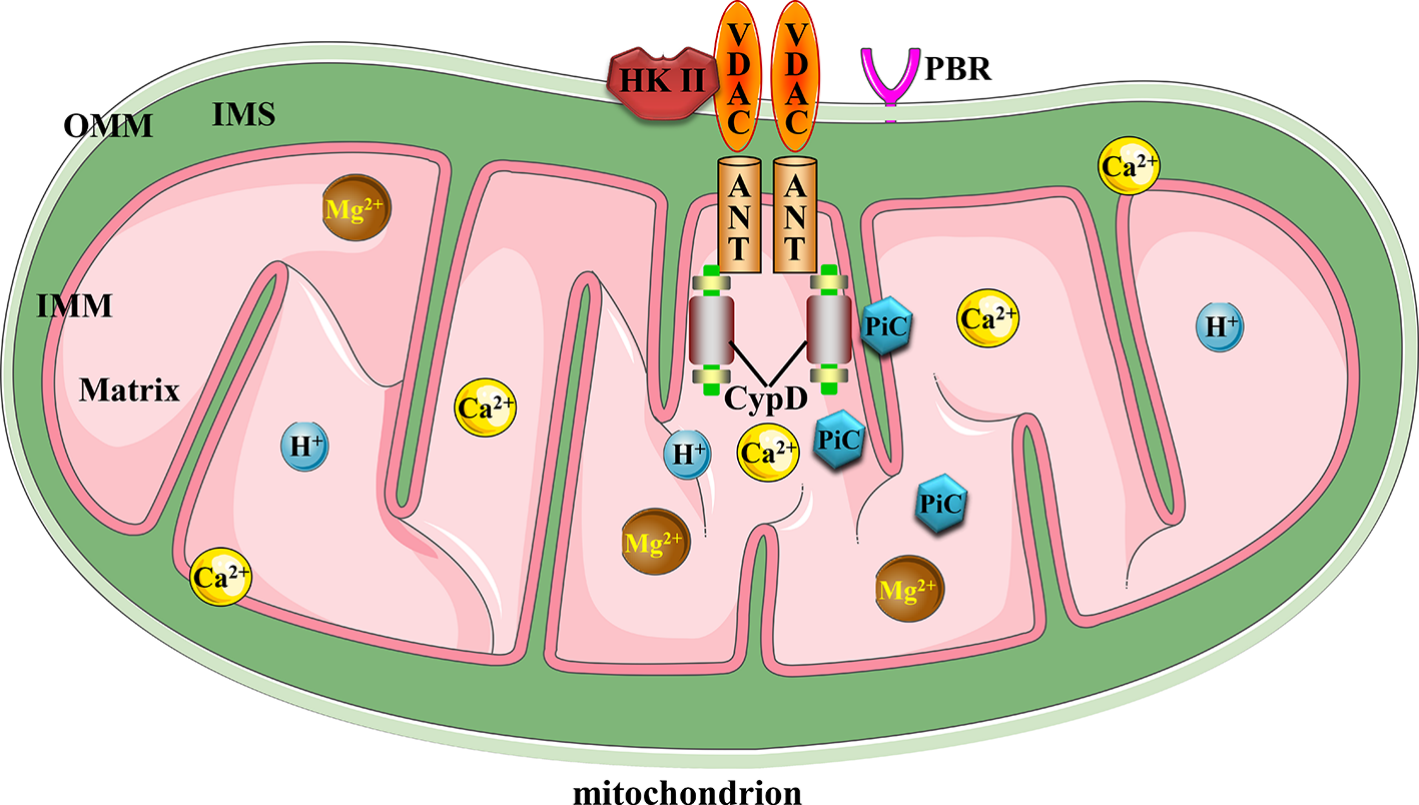
Canonical mitochondrial MPTP molecular structure. Conventional MPTP complex is composed of VDAC, ANT, and CypD. Other factors could also stimulate MPTP opening.

The mitochondrial permeability transition pore (MPTP) complex is a non-specific and -selective channel composed of multiple proteins, which is voltage-dependent and spans cytoplasm, outer mitochondrial membrane (OMM), inner mitochondrial membrane (IMM), and mitochondrial matrix. Excessive MPTP opening has been reported in relation to myocardial ischemia reperfusion injury (Morciano et al., 2017), hepatic ischemia-reperfusion injury (Panel et al., 2019), traumatic brain injury (Hånell et al., 2015), premature aging (Zhou et al., 2019), and Parkinson’s disease (Ludtmann et al., 2018). However, its structural composition of MPTP (Baines and Gutiérrez-Aguilar, 2018) and detailed regulatory mechanism in ischemic stroke are still poorly understood. To our knowledge, current evidences support the fact that MPTP is composed of voltage-dependent anion channel (VDAC) across the OMM, adenine nucleotide translocator (ANT) in the IMM, and cyclophilin D (CypD) in the mitochondrial matrix, which is responsible for sensing intracellular environmental oxidative stress injury, inflammatory cascade, pH imbalance, and ion disorders such as Ca^2+^ and Mg^2+^ ions in response to tissue ischemia (Kalani et al., 2018; Briston et al., 2019). These adverse factors, alone or together, can force persistent and irreversible MPTP opening beyond the range of physiological regulation, and thus inducing mitochondria-dependent apoptotic events. In addition, cytoplasmic hexokinase II (HK II) attached to VADC, the peripheral benzodiazepine receptor (PBR) on OMM and creatine kinase responsible for ATP production may be involved in the formation or regulation of MPTP (Zamzami and Kroemer, 2001). Possibly as a component of IMM and binding partner of CypD, the phosphate carrier (PiC) of mitochondria is responsible for the supply of inorganic phosphates required by ATP synthesis during oxidative phosphorylation of mitochondria (Brenner and Moulin, 2012; Bernardi et al., 2015; Solesio et al., 2016). However, whether PiC has a positive or negative effect on the structure and function of MPTP, it is still a matter of debate and disagreement. **Figure 1** illustrates the canonical molecular composition of MPTP.

In recent years, several other members involved in MPTP regulation responsible for cell fate decision have also been identified in succession. As one of the core components of IMM, RNAi-targeted silencing of the spastic paraplegia 7 gene blocked signal transmission between OMM and mitochondrial matrix by indirectly associating VDAC with CypD in the matrix, thereby abrogating overloaded Ca^2+^ and immoderate ROS evoked mitochondrial membrane potential (MMP) decline and MPTP-dependent cell death (Shanmughapriya et al., 2015). Except for the re-confirmation that ANT was the basic intimal component of MPTP, researchers had also found that other CypD-dependent components were involved in the composition of MPTP. Although the species destined for existence had not yet been identified, the authors suggested that PiC such as the *Slc25a3* or F_1_F_O_ ATP synthase may be involved, which still needed to be explored in a reasonable and rigorous *in vivo* and *in vitro* experiment (Karch et al., 2019). Further encouraging evidence suggested that F-ATP synthase was involved in the formation of MPTP, sensing Ca^2+^ concentration and subsequently mediating MPTP opening (Urbani et al., 2019). As the structure and functions of MPTP are gradually clarified, great quantities of studies have declared that abnormal MPTP conditions play a critical role in regulating cell fate in a variety of diseases. The VDAC has virtually no barrier effect on small molecules with molecular weights less than 5 kDa to circulate freely in the cytoplasm and mitochondrial matrix (Bonora and Pinton, 2019). As an intermediate bridge, ANT can interact directly with VDAC and CypD. And that is, ANT can alter OMM and IMM permeability by regulating VDAC and CypD, thus mediating the exchange of substances in the cytoplasmic matrix and the mitochondrial matrix (Chinopoulos, 2018). In a mouse model of heart failure, it had been substantiated that increased mitochondrial matrix Ca^2+^ caused by *Ppif* gene (encoding the synthesis of CypD protein) deficiency contributed to the remission of heart failure symptoms (Elrod et al., 2010). By further silencing CypD gene with *in vitro* siRNA and shRNA techniques on primary human pulmonary artery endothelial cells, and *in vivo* CypD knockout mice, evidence of CypD deficiency had been shown to promote angiogenesis, which may be partly due to increased mitochondrial matrix Ca^2+^ and nicotinamide adenine dinucleotide (NADH), activation of NAD^+^-dependent deacetylase sirtuin 1 (SIRT1) and serine-threonine kinase Akt signaling (Marcu et al., 2015). Evidence suggested that induced pluripotent stem cells (iPSCs) derived hepatocyte toxicity caused by valproic acid was associated with MPTP opening dependent mitochondrial apoptotic pathway (Li et al., 2015).

### Causality Between Abnormal MPTP Opening and Apoptosis in Ischemic Stroke

Abnormalities of MPTP state are bound to trigger cellular dysfunction in ischemic stroke. We will briefly summarize the factors and related molecular mechanisms of MPTP opening-induced apoptosis after ischemic stroke. A large number of previous reports have shown that stroke-evoked decreased MMP, excessive mitochondrial reactive oxygen species (mtROS) (Zorov et al., 2014), endoplasmic reticulum stress (ERS), and excitatory amino acid toxicity all stimulated MPTP opening (Prentice et al., 2015), leading to mitochondrial edema, increased membrane permeability, corrupted cristae structure of IMM, and neuronal apoptosis. Notably, as the second messenger, Ca^2+^ is a stimulus of MPTP opening and also could be a landmark event after MPTP opening. However, from the actual effect, increased Ca^2+^ and depressed matrix Mg^2+^ and Mn^2+^ could all contribute to MPTP opening. In turn, evidence had announced that instantaneously MPTP opening could cause increased Ca^2+^ in microdomain of astrocytes, which was closely related to maintaining mitochondrial energy supply and stress response (Agarwal et al., 2017). The otherwise MPTP opening-prone factors are as following. Declined matrix PH, caused by protonation of histidine residues or loss of ANT and CypD signaling, could trigger MPTP to tend to shut down. Conversely, the increased matrix PH forces MPTP opening with its maximum openness at about 7.3 (Wang et al., 2016; Šileikytė and Forte, 2019). The formation of disulfide by oxidation on ANT dimer, oxidized pyridine nucleotides such as NAD^+^ and NADP^+^ all favor MPTP openness. Conversely, all the factors that inhibit MPTP opening may have a promising future in treating ischemic stroke. Ligands targeting VADC, ANT, CypD (Matsumoto et al., 1999), and TSPO/PBR targets have shown better inhibition of MPTP opening. Moreover, antioxidants such as propofol, metabolites such as glucose and creatine, coenzyme Q, glutamate, or Ca^2+^ chelators could limit MPTP opening (Zamzami and Kroemer, 2001; Brenner and Moulin, 2012).

It is well known that onset of ischemic stroke causes neurons to produce exorbitant mtROS, ERS, Ca^2+^ overload, and neuronal toxicity induced by excitatory amino acids. After that, neurons would raise the alarm of MMP decline, mitochondrial edema, elevated MMP and other signs of MPTP opening, which will eventually drive mitochondrial contents such as Cyto-c to be discharged into the cytoplasm and trigger apoptotic events. The results of *in vivo* animal evaluation have intimated that both transient and permanent cerebral ischemic insults can cause damage to mitochondrial ultrastructure of neuron, such as the appearance of swollen and condensate mitochondria, as well elevated matrix density caused by deposition of electron-dense material (Solenski et al., 2002). An ischemia-induced ROS elevation can favor MPTP opening, which in turn can lead to a subsequent surge in ROS production and a vicious cycle (Zorov et al., 2014). Therefore, inhibition of neuronal apoptosis by blocking MPTP opening would be a potential and promising strategy in the treatment of ischemic stroke. Further extensive *in vivo* and *in vitro* experimental evidence also suggested a positive effect of this therapy. In rat models of ischemic stroke, blocking MPTP opening by cyclosporine A had been shown to reduce infarcted volume of ischemic brain tissue (Matsumoto et al., 1999). As a ligand targeting CypD, pre-administration of cyclosporine A can protect primary rat neurons from OGD/R injury, involved mechanisms may be related to maintain mitochondrial integrity and inhibit MPTP opening-induced apoptosis by up-regulating Parkinson’s disease-associated protein DJ-1 (Tajiri et al., 2016). Further, the water-soluble coenzyme Q10 had been shown to protect the accumulation of glutamate-induced HT22 hippocampal neuron damage by inhibiting mitochondrial fragmentation and MPTP opening-induced apoptosis (Kumari et al., 2016). Furthermore, evidence had shown that intervention of MPTP opening inhibitor can reduce the expression of VDAC, manifesting by increased MMP, ATP supply, and improved cerebral ischemia injury symptoms in an *in vitro* rat model of MCAO (Wang et al., 2019a). The above evidence all conveys that ischemic stroke induced MPTP opening may be a factoid of neuronal apoptosis. Any measures to inhibit MPTP opening could repress cell apoptosis, thus exhibiting the role of anti-ischemic brain protection.

Explosive evidence corroborated that a sudden insult of ischemic stroke may break the balance between the anti-apoptotic and pro-apoptotic members of B-cell lymphoma-2 (Bcl-2) family, which may aggravate stroke condition. The results of ischemic stroke models with Bax gene knockout *in vivo* and *in vitro* showed that the improved ischemic neuron injury and decreased neuronal apoptosis were related to the decreased cytoplasmic Ca^2+^, which was a relatively upstream signal regulating the apoptosis of ischemic neurons (D’Orsi et al., 2015). A great deal of evidence has declassified such a fact that anti-apoptotic Bcl-2 and Bcl-x_L_ can inhibit MPTP opening, whereas pro-apoptotic Bax and Bak proteins can stimulate MPTP opening (D’Orsi et al., 2017). Results from *in vitro* model of ischemic stroke in rats have shown that increased Bax/Bcl-2 ratio in ischemic insult could irritate MPTP opening, which may cause increased neuronal apoptosis (Andrabi et al., 2017; Andrabi et al., 2019). Actually, members in Bcl-2 family could also regulate two potential MPTP opening stimuli: Ca^2+^ homeostasis and energy metabolism of neurons (D’Orsi et al., 2017; Peña-Blanco and García-Sáez, 2018). The increased permeability caused by the formed Bax/Bak dimer on OMM contributed to the release or transfer of pro-apoptotic Cyto-c, Smac/Diablo and HtrA2/Omi from the mitochondrial matrix to cytoplasm (Arnoult et al., 2003). Further rat models of focal cerebral ischemia also demonstrated that overexpressed Bcl-2 protein could inhibit the rise of Cyto-c in cytoplasm, thereby preventing the occurrence of apoptotic DNA fragmentation events mediated by the transfer of AIF from mitochondria to nucleus (Zhao et al., 2004). While pro-apoptotic Bcl-x_S_-induced apoptosis via Bak also induced the exudation of mitochondrial Cyto-c, the formation of apoptosome composed of Cyto-c, Apaf-1 and Caspase-9, and the Caspase apoptotic cascade (Lindenboim et al., 2005; Zhang Q. et al., 2019). Visually breaking news, Bak/Bax macropores contribute to the outflow of mitochondrial contents such as Cyto-c and mitochondrial DNA into the cytoplasm, and thereafter inducing caspase-dependent cell apoptosis (McArthur et al., 2018). Amusingly, evidence also suggested that ROS and ERS could directly activate Bak/Bax-dependent apoptosis, showing condensed and hyperchromatic nucleus, loss of MMP, reduced Bcl-2, increased activation of Caspase-3/-9, PARP, and overexpressed Bak and Bax proteins (Seervi et al., 2018). Therefore, the formation of Bak/Bax macropores in mitochondrial OMM may serve as a hub for MPTP opening-induced mitochondrial apoptosis. Other factors involved in the regulation of MPTP opening after cerebral ischemia have also been reported. Accumulation of p53 in mitochondria has been corroborated to target CypD, leading to MPTP opening and neuronal apoptosis, which is independent of the formation of Bak/Bax macropores (Vaseva et al., 2012). It has been reported that activation of neuron mitochondrial cannabinoid receptor 1 after cerebral ischemia can help inhibit Ca^2+^ overload-induced MPTP opening and apoptosis (Cai et al., 2017). Another potential target involved in regulating mitochondrial MPTP in ischemic stroke was mitochondrial uncoupling protein 2 (UCP-2). Highly expressed UCP-2 has been demonstrated to inhibit apoptosis by activating redox signaling, evidenced by decreased ROS production, increased MMP and cleaved Caspase-3 protein expression (Mattiasson et al., 2003; Mehta and Li, 2009). The above analysis indicates that ischemic stroke is accompanied by an inevitable event of MPTP over-opening and apoptosis. Although the basic structure of MPTP has not yet been drastically uncovered and recognized. But a number of factors that regulate MPTP opening during the course of ischemic stroke have been exposed in the public eye. In the future, plenty of basic studies should be conducted to elucidate the molecular composition of MPTP and its relationship with ischemic neuron apoptosis. Meanwhile, natural product inhibitors targeting MPTP opening-evoked neuronal apoptosis are also worthy of further research in the treatment of ischemic stroke.

## Progress in Stroke Prevention and Treatment by Regulating Mitochondrial MPTP Status in Ethnic Medicine

Ischemic stroke, which accounts for 71% of stroke, is the second and the first leading cause of death and disability worldwide and in China, respectively (Wu S. et al., 2019). In 2016, there were 9.5 million ischemic stroke patients worldwide, while in 2017, 2.7 million people died of ischemic stroke (Campbell et al., 2019). Although intravenous thrombolysis, antiplatelet aggregation, and anticoagulant therapy (Smith et al., 2019; Stoll and Nieswandt, 2019) could be used for the delivery of stroke therapies, but many apoplexy sequelae, characterized by ischemic contralateral or bilateral limb behavior disorders, memory decay, logopathy, dysphagia, and mood irritability (Zhao et al., 2016; Hou et al., 2020), have not yet cure. However, the ethnic medicine has manifested significant clinical efficacy in alleviating above unbearable symptoms or sequelae of stroke. In recent years, the mechanisms of action of drugs have also been gradually revealed. Remarkably, some of them, such as Danhong injection and Naoxintong capsule (Haiyu et al., 2016; Liu M. et al., 2016; Xu et al., 2020), have been officially approved by the China Food and Drug Administration (CFDA) and are bringing good news to stroke patients around the world. Next, we summarized the current officially authorized products, clinically effective traditional Chinese medicine (TCM) prescriptions, ethnic drugs, and effective monomer components based on literature review, trying to clarify the molecular mechanisms of natural products inhibiting neuronal apoptosis and improving ischemic brain from the perspective of mitochondrial MPTP.

### Authorized Products for Stroke Improvement by Regulating Mitochondrial MPTP

With the policy guidance and inclination, as well as the accelerated modernization of TCM, tens of thousands of individuals dedicated to clinical and scientific research positions are gradually devoting themselves to the drug development and mechanism exploration of traditional medicine to prevent major diseases, such as stroke. Most ethnic drugs for treatment of ischemic stroke have the function of activating blood circulation to remove blood stasis or clear collaterals. NaoShuanTong capsule (Zhang H. et al., 2019), ShenQi Fuzheng injection (Cai et al., 2016), ShengMai injection (Yang et al., 2016), and PeiYuan TongNao capsule (Bai J. et al., 2019) have been reported to significantly improve the symptoms of ischemic stroke with few adverse events. In recent years, some antiapoptotic protective effects of cerebral ischemia have also been reported, such as XueShuanTong injection (Li et al., 2009) and QianCao NaoMaiTong mixture (Lu et al., 2016).

Most, such as Cerebralcare Granule^®^ (Sun et al., 2010), DanHong injection (Xu J. et al., 2017; Li M. et al., 2018), and AnGong NiuHuang wan (Wang G. et al., 2014; Tsoi et al., 2019), can inhibit ischemia-evoked neuronal apoptosis by regulating bcl-2 family members. As a prescription commonly used in Tibetan medicine to treat ischemic sequelae, our research group proved that the anti-cerebral ischemia effect of ErShiWei ChenXiang pills may be related to its regulation of Bcl-2 family, inhibition of apoptosis, and increase of energy supply (Hou et al., 2020). While regulating Bcl-2 family members, AnNao tablets (Zhang et al., 2020) and YiQi FuMai powder injection (Cao et al., 2016; Xu Y. et al., 2017) may also be involved in inflammation and mitochondrial autophagy to maintain mitochondrial MMP and energy production. In addition, both TongXinLuo's regulation of AKT/ERK signaling (Yu et al., 2016; Cheng et al., 2017) and XingNaoJing injection's regulation of the PI3K-AKT pathway (Zhang Y. et al., 2018) ultimately contributed to the regulation of Bcl-2 and the inhibition of ischemic neuron apoptosis. In addition to the Bcl-2 family, it was reported that Zhenlong Xingnao capsule (Wei X. et al., 2019) and NaoLuoTong capsule (Bai M. et al., 2019) could also be through the regulation of NF-кB to confine ischemia induced inflammatory cascade process. Of course, multiple mechanisms of drugs have also been reported against ischemic neuron apoptosis. QingKaiLing injection could simultaneously inhibit oxidative stress, activation of NLRP3 inflamosome and AMPK signaling pathway, and thus inhibiting neuronal apoptosis (Cheng et al., 2012). PienTzeHuang capsule suppressed the inflammatory and apoptotic cascade of ischemia by regulating AKT/GSK-3β and the Bcl-2 family (Zhang X. et al., 2018). As a fatal blow to the body, disregardful ischemic stroke induced hypoimmunity was also one of the main culprits of exacerbating stroke. Noteworthy, XueSaiTong (Li et al., 2019) and Danggui-Jakyak-San (Kim et al., 2016) may mediate inflammatory responses by regulating STAT3 signaling pathway, and enhance immune function of the body, which were helpful to reduce symptoms of brain injury after ischemia. The above officially certified drugs’ information and specific mechanisms of action are shown in **Supplementary Table 1**, and **Tables 1** and **2**. Through in-depth comparative analysis, we found that although the above drugs prevailing in the market have good clinical efficacy, most of their active ingredients, *in vivo* pharmacokinetic parameters, and potential targeted organ toxicity have not been well evaluated. Importantly, the further regulation of apoptosis still has good research value and prospect. Although there is no direct evidence that they regulate MPTP to inhibit ischemic neuron apoptosis, their effect on members of the Bcl-2 family makes MPTP a potential target for anti-stroke drugs.

**Table 1:** The *in vivo* mechanism underlying the inhibition of MPTP opening-induced neuronal apoptosis by authorized drugs in the treatment of ischemic stroke.

Agents	Objects	Gender	Weight (g)	Animal model	Dose	Time periods	Mechanisms	References
XueShuanTong injection	SD	Both	270–320	MCAO (2 h)/R (46 h)	25 mg/kg, i.p.	Pretreatment for 5 min and 12/24/36 h after MCAO	Caspase-1/3↓, TUNEL-positive neurons↓	Li et al., 2009
Cerebralcare granule	Mongolian gerbils	Male	65–90	MCAO (0.5 h)/R (5 d)	0.4 and 0.8 g/kg, i.g.	3 h after the reperfusion, 5 d, q.d.	Bcl-2↑; leukocyte adhesion↓, fluorescence intensity of DHR↓, albumin leakage↓, Caspase-3↓, Bax↓, TUNEL-positive neurons↓	Sun et al., 2010
AnGong NiuHuang wan.	SD	Male	250–280	MCAO (1.5 h)/R (24 h)	0.065, 0.125, and 0.25 g/kg, i.g.	Pretreatment 3 d, q.d., and 1 d, q.d. after reperfusion	Bcl-2↑; Bax↓, Caspase-3↓, TUNEL-positive neurons↓	Wang G. et al., 2014
	SD	Male	260–280	MCAO (2 h)/R (22 h)	257 mg/kg, i.g.	Single dose before reperfusion	Bcl-2↑, ZO-1↑, claudin-5↑, eNOS↑; Bax↓, p47_phox_↓, iNOS↓, 3-NT↓, MMP-2↓, MMP-9↓, iNOS↓	Tsoi et al., 2019
QingKaiLing injection	KM/C57BL/6	Male	25–28/25–30	MCAO (1.5 h)/R (28 h)	3 ml/kg, i.v.	4 h after reperfusion, and once every 12 h, three times	Procaspase-12↑; Caspase-3↓, p-eIF2α↓, ROS↓, Ca^2+^↓, TUNEL-positive neurons↓	Cheng et al., 2012
DangGui Jakyak san	SD	Male	——	pMCAO (28 d)	50, 100, and 200 mg/kg, i.g.	24 h after surgery, 28 d, q.d.	STAT3↑, Pim-1↑, GSH↑, SOD↑, CAT↑; MDA↓, Caspase-3↓, PARP↓, NT↓, 4-HNE↓	Kim et al., 2016
YiQi FuMai powder injection	C57BL/6J	Male	18–22	pMCAO (24 h)	1.342 g/kg, i.p.	Single dose after pMCAO onset	cerebral blood flow↑, Bcl-2↑; Caspase-12↓, GRP78↓, CHOP↓, ATF-4/6↓, p-eIF2*α*/eIF2*α*↓, XBP-1↓	Cao et al., 2016
	SD	Male	280–300	tMCAO (1.5 h)/R (24 h)	0.957 g/kg, i.p.	Single dose after tMCAO onset	Bcl-2↑, cytosolic Drp1↑; Bax↓, cleaved Caspase-9↓, mtDrp1↓, total p-Drp1 and Drp1↓	Xu Y. et al., 2017
TongXinLuo	SD	Male	200–220	MCAO (1.5 h)/R (24 h)	0.4, 0.8, and 1.6 g/kg, i.g.	Pretreatment for 3 d, b.i.d., and after MCAO for 1 d, b.i.d.	p-PTEN/PTEN↑, p-PDK1/PDK1↑, p-AKT/AKT↑, p-Bad/Bad↑, p-c-Raf/c-Raf↑; cleaved Caspase-3↓, TUNEL-positive neurons↓	Yu et al., 2016
	SD	Male	240–270	MCAO (1.5 h)/R (14 d)	0.1 g/kg, i.g.	Pretreatment for 5 d and 14 d after MCAO, q.d.	Connexin 43↓, Calpain II↓, Bax↓, cleaved Caspase-3↓, TUNEL-positive neurons↓	Cheng et al., 2017
QianCao NaoMaiTong mixture	SD		180–200	MCAO (2 h)/R	2.7, 5.4, and 10.8 ml/kg	Pretreatment for 28 d	Bcl-2/Bax↑, SOD↑, CAT↑, BDNF↑, ICAM-1↑, NGF↑, MDA↓, IL-6↓	Lu et al., 2016
DanHong injection	SD	Male	250–280	MCAO (1 h)/R (24 h)	4 ml/kg, i.p.	4 h after MCAO	claudin-5↑, occludin↑, ZO-1↑, Bcl-2↑; Bax↓, Caspase-3↓, MMP-9↓, PAI-1↓, P-selectin↓	Li M. et al., 2018
XingNaoJing injection	SD	Male	250–280	MCAO (2 h)/R (24 h)	5, 10, and 15 ml/kg, i.p.	24 h after reperfusion	Bcl_2_/Bax↑, p-PI3K/PI3K↑, p-AKT (308 and 473)/AKT↑, p-eNOS/ eNOS↑, NO↑, p-PI3K/AKT↑	Zhang Y. et al., 2018
PienTzeHuang capsule	SD	Male	240–260	MCAO (1.5 h)/R (24 h)	180 mg/kg, i.g.	Pretreatment 4 d before MCAO	NeuN↑, mtCyto-c↑, Bcl-xl↑, p-AKT↑, p-GSK-3β↑; IL-1β↓, IL-6↓, TNF-α↓, cytosolic Cyto-c↓, Bax↓, p53↓, cleaved Caspase-3/9↓, TUNEL-positive neurons↓	Zhang X. et al., 2018
XueSaiTong	C57BL/6	Male	20–25	MCAO (45 min)/R (14 d)	15 µg/g, i.v.	Immediately after reperfusion, 14 d, q.d.	arginase-1↑, CD206↑, CD206/Iba-1↑, IL-10↑, TGF-β1↑; IL-1β↓, p-STAT3/STAT3↓, CD16↓, CD16/Iba-1↓, iNOS↓, TUNEL-positive neurons↓	Li et al., 2019
NaoLuoTong capsule	Wistar	Male	250–280	MCAO (2 h)/R (22 h)	75, 150, and 300 mg/kg, i.g.	Pretreatment for 7 d, q.d.	Bcl-2↑, NGF↑; TNF-α↓, IL-1β↓, IL-6↓, Bax↓, Caspase-3↓, ICAM-1↓, NF-κBp65↓	Bai M. et al., 2019
ZhenLong XingNao capsule	Wistar	Male	200–250	MCAO (1.5 h)/R (24 h)	125 and 250 mg/kg, i.g.	Pretreatment 14 d, q.d.	T-AOC↑, T-SOD↑, Bcl-2↑, Bcl-2/Bax↑; Caspase-3↓, NF-кB↓, p38↓, Bax↓, MDA↓, GABA↓, Glu↓, Tau↓	Wei X. et al., 2019
ErShiWei ChenXiang pills	SD	Male	260–300	MCAO (2 h)/R (24h)	1.33 and 2.00 g/kg, i.g.	Pretreatment 14 d, q.d.	Bcl-2↑, CaMK II↑; Bax↓, cleaved Caspase-3↓, Cyto-c↓, ATF4↓, c-Jun↓, TUNEL-positive neurons↓	Hou et al., 2020
AnNao tablets	SD	Male	250–270	MCAO (2 h)/R (7 d)	300, 600, and 1,200 mg/kg, i.g.	1 h after reperfusion, 1 d or 7 d, q.d.	Drp1↑, OPA1↑, PINK1↑, Parkin↑, Bcl-2↑, Bcl-2/Bax↑; Bax↓	Zhang et al., 2020

↑, upgrade; ↓, downgrade.

**Table 2:** The *in vitro* mechanism underlying the inhibition of MPTP opening-induced neuronal apoptosis by authorized drugs in the treatment of ischemic stroke.

Agents	Cell lines	Model	Dose	Time periods	Mechanisms	References
TongLuo JiuNao injection	BMECs of SD rats	OGD (95% N_2_ and 5% CO_2_ 6 h)/R (74% N_2_, 21% O_2_, and 5% CO_2_, 6 h)	2 μl/ml	Before OGD, the neurons were incubated 6 h in drug treatment and then equilibrated OGD	VEGF↑, MMP↑; LDH↓, Ca^2+^↓, cytosolic Cyto-c↓, NMDAR1↓, PAF↓	Li et al., 2014
QianCao NaoMaiTong mixture	SH-SY5Y	OGD (N_2_, 1 h)/R (24 h)	0.5, 1, 5, 10, 50, 100 and 200 mg/ml	Pretreatment for 2 h and during reperfusion period	Caspase-3/8↓, neuronal apoptosis under flow cytometry↓	Lu et al., 2016
YiQi FuMai powder injection	PC12	OGD (5% CO_2_, 94% N_2_, and 1% O_2,_ 12 h)	100, 200, and 400 μg/ml	during OGD period	Bcl-2↑; neuronal apoptosis under flow cytometry↓, Caspase-3↓, cleaved Caspase-3↓, Caspase-12↓, CHOP↓, GRP78↓, ATF-4/6↓, p-eIF2*α*/eIF2*α*↓, XBP-1↓, Hoechst 33342 positive neurons↓	Cao et al., 2016
	PCN of embryonic, 16–18-d SD rats	100 μM H_2_O_2_ for 12 h	100, 200, and 400 μg/ml	6 h before and during H_2_O_2_ treatment	ATP↑, MMP↑; Bcl-2↑, Bcl-xl↑, cytosolic Drp1↑, cytosolic PKCδ↑; Bax↓, Bak↓, Caspase-3↓, cleaved Caspase-3↓, mtROS↓, PKCδ↓, neuronal apoptosis under flow cytometry↓, intracellular ROS↓, p-Drp1/Drp1↓, mtDrp1↓, mtPKCδ↓	Xu Y. et al., 2017
DanHong injection	PCN of embryonic, 14-d C57 BL/6 mice	OGD (95% N_2_ and 5% CO_2_, 6 h)	0.01, 0.03, 0.1, 0.3, and 1 μl/ml	During OGD period	LDH↓, ROS↓, Ca^2+^↓, neuronal apoptosis under flow cytometry↓	Xu J. et al., 2017
XingNaoJing injection	HBMECs	OGD (5% CO_2_, 85% N_2_, and 10% H_2,_ 3 h)/R (24 h)	1.5 and 2.5 *μ*l/ml	Pretreatment for 1 h and during reperfusion period	p-eNOS/eNOS↑, MMP↑, NO↑; cleaved Caspase-3/Caspase-3↓, neuronal apoptosis under flow cytometry↓	Zhang Y. et al., 2018

↑, upgrade; ↓, downgrade.

### Prescription and Molecular Mechanisms in Regulating MPTP Openness of Ischemic Stroke

Clinical experience has proved that TCM has excellent efficacy in treating stroke, which can be seen in *Huangdi Neijing*. But at bottom it is the cold, hot, warm, cool, and other characteristics of drugs to balance the imbalance of Yin and Yang in the body under the condition of disease. In ischemic stroke, a variety of exogenous pathogens and dysfunction of the viscera can lead to poor blood flow or blood stasis, resulting in cerebral ischemia or hypoxia (Hou et al., 2020). Therefore, the clinic mainly focuses on promoting blood circulation to remove stasis, replenishing Qi to nourish blood, and nursing viscera. Extensive clinical and *in vivo* and *in vitro* studies have confirmed that prescriptions SiJunZi decoction (Yang et al., 2019), ShengMai san (Li et al., 2013), and YangYin TongNao granules (Wang et al., 2019f) have a significant effect on ischemic stroke. Of course, the regulation of oxidative stress and inflammatory response are also common mechanisms of prescription in the treatment of ischemic brain injury. The antioxidant and anti-inflammatory activities of ShengNaoKang decoction (Chen et al., 2014) could contribute to the inhibition of apoptosis and the alleviation of ischemic brain injury. Other studies have reported that HuangLian JieDu decoction (HJD) could inhibit ischemic neuron apoptosis by regulating PI3K/AKT and HIF-1α/VEGF (Zhang Q. et al., 2014). Further metabolomics (Zhu et al., 2018) and systemic pharmacology (Wang P. et al., 2019) studies have revealed that its anti-ischemic protective effect may also involve the Bcl-2 family such as Bak. Regulating vascular function and increasing cerebral blood flow supply is another effective strategy for stroke treatment. Abundant evidence demonstrated that BuYang HuanWu decoction (BHD) could increase cerebral blood by regulating HIF-1α/VEGF-related signaling pathways (Chen et al., 2019). Improving the mitochondrial ATP supply has also been shown to be an effective treatment for stroke. BHD has been reported to improve ischemic brain injury by reducing glutamate-mediated excitatory amino acid toxicity, resulting in enhanced ATP supply and weakened apoptosis (Wang et al., 2011). At the same time, the improved synaptic ultrastructure by BHD also contributed to the recovery of cerebral ischemia sequelae (Pan et al., 2017). Similarly, ShenGui SanSheng san could also improve the efficiency of citric acid cycle to improve the brain energy deficit after ischemia (Luo et al., 2019). Interestingly, as a cell-sensing oxygen sensor, most studies have also reported evidence of other TCM prescriptions regulating HIF-1α to inhibit apoptosis and inflammation in treatment of stroke, such as XueFu ZhuYu decoction (Lee et al., 2011) and TaoHong SiWu decoction (Yen et al., 2014). Members of the Bcl-2 family are also potential targets for prescription inhibition of apoptosis to improve ischemic brain injury. XiaoXuMing decoction (Lan et al., 2014), ShuanTongLing (Mei et al., 2017), and GuaLou Guizhi decoction (Zhang Y. et al., 2014) all have the potential to regulate the Bcl-2 family and inhibit caspase-dependent mitochondrial apoptosis, which has a similar mechanism to that of MuXiang You fang (Zhao et al., 2016) reported in our previous study. In addition to the Bcl-2 family, DiHuang YinZi (Hu et al., 2009) and DiDang tang (Huang et al., 2018) could also inhibit the generation of Ca^2+^ and improve MMP to inhibit the apoptosis of ischemic neurons by regulating the ERK signaling pathway. It has also been reported that HouShiHei san (Chang J. et al., 2016) could regulate PI3K/Akt signaling to inhibit the apoptosis of ischemic neurons. The specific mechanisms *in vivo* and *in vitro* of the above prescriptions are shown in **Table 3**.

**Table 3:** The *in vivo* and *in vitro* mechanism underlying the inhibition of MPTP opening-induced neuronal apoptosis by TCM prescriptions in the treatment of ischemic stroke.

*In vivo* study								
Agents	Objects	Gender	Weight (g)	Animal model	Dose	Time periods	Mechanisms	References
DiHuang YinZi	Wistar	Both	320–350	MCAO (1 h)/R (10 d)	6 and 12 g/kg, i.g.	30 min after MCAO, 10 d, q.d.	synaptophysin↑, ERK↑; LDH↓, TUNEL-positive neurons↓	Hu et al., 2009
XueFu ZhuYu decoction	Wistar	Male	250–300	MCAO (1 h)/R (24 h)	1.5 and 3.0 g/kg, i.g.	Pretreatment for 14 d, q.d.	cleaved Caspase-3↓, HIF-1α↓, TNF-α↓, iNOS↓	Lee et al., 2011
BuYang HuanWu decoction	ICR	Male	17–22	MCAO (30 min)/R (14 d)	0.5 and 1.0 g/kg, i.g.	2 h after reperfusion, 14 d, b.i.d.	glucose metabolism↑, BrdU↑; ROS↓, TUNEL-positive neurons↓, CD11b↓	Wang et al., 2011
ShengNaoKang decoction	SD	Male	280–320	MCAO (2 h)/R (24 h)	0.7, 1.4, and 2.8 g/kg, i.g.	Pretreatment for 6 d and 1 d after reperfusion, q.d.	SOD↑; GSH-Px↑, Caspase-3↓, MDA↓, iNOS↓, TNOS↓	Chen et al., 2014
TaoHong SiWu decoction	Wistar	Male	250–300	MCAO (1 h)/R (24 h)	0.7 g/kg, i.g.	Pretreatment for 14 d, q.d.	cleaved Caspase-3↓, HIF-1α↓, iNOS↓, TNF-α↓	Yen et al., 2014
HuangLian JieDu tang	SD	Male	300–350	MCAO (2 h)/R (72 h)	2.7 g/kg, i.g.	Single dose and pretreatment for 24 h	p-PI3K/PI3K↑, p-AKT/AKT↑, HIF-1α↑, EPO↑, VEGF↑, BrdU↑; LDH↓, TUNEL-positive neurons↓	Zhang Q. et al., 2014
XiaoXuMing decoction	SD	Male	250–280	MCAO (1.5 H)/R (24 h)	60 g/kg, i.g.	Pretreatment for 3 d, t.i.d.	mtBcl-2↑, mtCyto-c↑, cytoplasmic Bax↑, cytoplasmic c-IAP1↑; mtbroken cristae↓, cleaved Caspase-3/9↓, p53↓, mtp53↓, mtBax↓, cytoplasmic Smac↓, cytoplasmic Cyto-c↓, TUNEL-positive neurons↓	Lan et al., 2014
GuaLou Guizhi decoction	SD	Male	280–300	MCAO (2 h)/R (7 d)	14.4 g/kg, i.g.	Posttreatment for 7 d, q.d.	NeuN↑, MAP-2↑, Bcl-2↑; GFAP↓, Bax↓, TUNEL-positive neurons↓	Zhang Y. et al., 2014
MuXiang You fang	SD	Male	260–300	MCAO (2 h)/R (48 h)	58, 116, and 232 mg/kg, i.g.	Posttreatment for 3 d, q.d.	Bcl-2↑, Bcl-2/Bax↑; Bax↓, Cyto-c↓, Caspase-3/7/9↓	Zhao et al., 2016
ShuanTongLing	SD	Male	250–280	MCAO (1.5 h)/R (24 h)	5.7 and 17.2 ml/kg, i.g.	Pretreatment for 7 d, q.d.	SIRT1↑, Bcl-2↑; TNF-α↓, IL-1β↓, Ac-p53↓, Bax↓	Mei et al., 2017
*In vivo* study								
Agents	Cell lines	Model	Dose	Time periods	Mechanisms			References
DiDang tang	PC12	OGD (95% N_2_ and 5% CO_2_, 0.5–2.5 or 2–10 h)	12.5, 25, and 50 mg/ml	After the OGD induced PC12 cell model for 24 or 48 h	Bcl-2/Bax↑; Ca^2+^↓, MMP↓, GRP78↓, p-IRE1/IRE1↓, p-PERK/PERK↓, p-eIF2*α*/eIF2*α*↓, p-Bad/Bad↓, ATF-6↓, Cyto-c↓, cleaved PARP↓, neuronal apoptosis under flow cytometry↓			Huang et al., 2018

↑, upgrade; ↓, downgrade.

The above evidence indicates that most TCM prescriptions could more or less improve mitochondrial morphology and respiratory function by inhibiting neuronal Ca^2+^ overload through anti-oxidative stress and anti-inflammatory. Meanwhile, we note that most of them also regulates many members of the Bcl-2 family to inhibit ischemic neuron apoptosis. We, therefore, see the potential of drugs to indirectly inhibit MPTP opening to improve ischemic neuron apoptosis. Nevertheless, the unclear drug distribution of target organs and the intricate network of interactive targets should still drive us to further study.

### Herbal Extracts and Molecular Mechanisms in Regulating MPTP Openness of Ischemic Stroke

**Figure 2:**
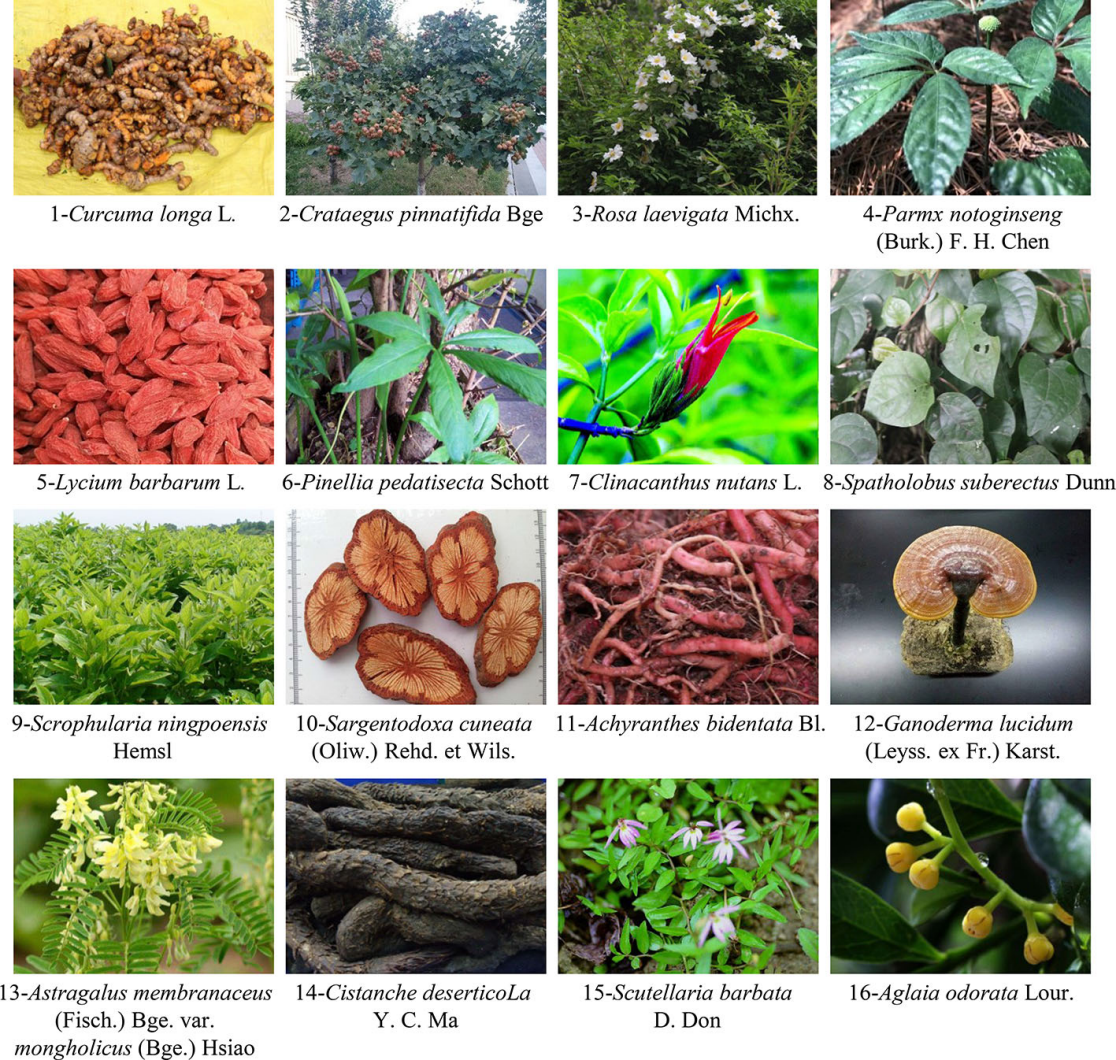
Representative herbal images that may inhibit ischemic neuron apoptosis by regulating MPTP. Sixteen herbs are shown here.

The overall concept of TCM and the characteristics of treatment based on syndrome differentiation of ethnic medicine determine that prescriptions from diversified drug sources are mainly used in the treatment of diseases. The purpose is to comprehensively consider the functions of viscera to exorcize evil spirits while strengthening the body, and finally cure diseases. However, in addition to conventional prescriptions mentioned above, people have also discovered that the individual application of certain herbs also has the potential to treat diseases. Based on recent literature reports, most of them exhibit outstanding antioxidant effects, such as methanol extract of *Artemisia absinthium* (Bora and Sharma, 2010) and *Colebrookea oppositifolia* Smith (Viswanatha et al., 2018). As the most sensitive hippocampal neuron to ischemic invasion, studies have shown that *Moringa oleifera* seed extract could promote hippocampal nerve regeneration, enhance synaptic plasticity and cholinergic function to treat ischemic stroke (Zeng et al., 2019). More interestingly, *Gynostemma pentaphyllum* extract could protect OGD/R-induced rats isolated hippocampal slices damage by inhibiting neuronal Ca^2+^ overload and mitochondrial oxidative stress-induced MPTP opening (Schild et al., 2009), which may help to inhibit the MPTP opening-activated mitochondrial apoptotic cascade event. At the same time, herbs could regulate the expression level of anti-apoptotic and pro-apoptotic proteins of Bcl-2 family and inhibit mitochondrial apoptosis in the treatment of hypoxia brain injury. The specific *in vivo* and *in vitro* mechanisms of reported herbs for ischemic stroke treatment by inhibiting mitochondrial MPTP opening-induced neuronal apoptosis are shown in **Tables 4** and **5**. **Figure 2** shows pictures of 16 representative herbs. It is world-renowned that superior immune enhancement of plant polysaccharides could prevent and cure many diseases. Previous investigations reported the anti-ischemic effects of *Ganoderma lucidum* polysaccharides (GLP) (Zhou et al., 2010), *Lycium barbarum* polysaccharide (LBP) (Wang T. et al., 2014; Zhao et al., 2017b), *Panax notoginseng* polysaccharides (PNP) (Dong et al., 2014), and *Cistanche deserticola* polysaccharides (CDP) (Liu et al., 2018) were associated with anti-oxidant activity and the regulation of Bcl-2 family members to maintain mitochondrial function and morphology. Furthermore, *Achyranthes bidentata* polypeptides (ABP) (Shen et al., 2010), astragalosides (Chiu et al., 2014), and phenolic acid extracts derived from *Sargentodoxa cuneata* (Bai M. et al., 2019) and *Salvia miltiorrhiza* (Hou et al., 2016; Yang et al., 2018; Wei Y. et al., 2016) also have potential anti-ischemic stroke effects. In conclusion, although the clinical treatment of ischemic stroke with a single herb is rare, a large number of definitive *in vitro* and *in vivo* and clinical reports are sufficient to support further studies. However, the mechanism of some herbs with better efficacy proved by experiments is still in the preliminary stage, and the ischemic brain protection mechanism of anti-neuronal apoptosis is worthy of further exploration. More promisingly, some ethnic herbs for stroke prevention, such as Tibetan medicine saffron (Ochiai et al., 2007) and Mongolian medicine Eerdun Wurile (Gaowa et al., 2018), have also been gradually reported in recent years. In the early stage, our research group also revealed that the anti-hypoxia brain protection effect of the Tibetan medicine *Rhodiola crenulata* was related to the regulation of the HIF-1α/microRNA 210/ISCU1/2(COX10) signal pathway to improve mitochondrial energy metabolism, inhibit oxidative stress and mitochondrial apoptosis (Wang et al., 2019c). Although the medication law of ethnic medicine for prevention and treatment of ischemic stroke is bound to limit the scope of effective single herbal medicine. But optimistically taking the long view, such a gradual herbal medicine research model should be warranted.

**Table 4:** The *in vivo* mechanism underlying the inhibition of MPTP opening-induced neuronal apoptosis by herbal medicine in the treatment of ischemic stroke.

Agents	Objects	Gender	Weight (g)	Animal model	Dose	Time periods	Mechanisms	References
Curcuma oil	SD	Male	200–225	MCAO (1 h)/R (24 h)	250 mg/kg, i.p.	Single dose and pretreatment for 0.5 h	Bcl-2↑, MMP↑; MPO↓, nitrite↓, nitrate↓, iNOS↓, nNOS↓, e NOS↓, peroxynitrite↓, ROS↓, Ca^2+^↓, Cyto-c↓, p53↓, cleaved Caspase-3, Bax↓, TUNEL-positive neurons↓, neuronal apoptosis under flow cytometry↓	Dohare et al., 2008
Hawthorn extract	SD	Male	300–320	MCAO (1.25 h)/R (3 or 24 h)	100 mg/kg, i.g.	Pretreatment for 15 d, q.d.	Bcl-xL↑, Foxp3↑, pSTAT-3/STAT-3↑; IL-10↑; MPO↓, TNF-α↓, IL-6↓, IL-1β↓, ICAM-1↓, CD3^+^ & CD8^+^ positive cells↓, TUNEL-positive neurons↓	Elango and Devaraj, 2010
*Rosa laevigata* Michx	SD	Male	250–300	MCAO (2 h)/R (24 h)	50, 100, and 200 mg/kg, i.g.	Pretreatment for 7 d, q.d.	Bcl-2↑, SOD↑, GSH↑, MMP↑; MDA↓, T-NOS↓, NO↓, iNOS↓, MMP-9↓, p53↓, Apaf1↓,Fas↓, Fasl↓, Bax↓, Bid↓, Cyto-c↓, cleaved Caspases-3/8/9↓, NF-κB↓, COX-2↓, TNF-α↓, IL-1β↓, IL-4↓, IL-6↓, p-JNK↓, p-ERK↓, p-p38↓, TUNEL-positive neurons↓	Zhang et al., 2013
PNP	Wistar	Male	250–300	MCAO (2 h)/R (22 h)	50, 100, and 200 mg/kg, i.g.	Pretreatment for 7 d, q.d.	Bcl-2/Bax↑; cleaved Caspase-3↓, TUNEL-positive neurons↓	Dong et al., 2014
LBP	ICR	Male	20–25	MCAO (2 h)/R (24 h)	10, 20, and 40 mg/kg, i.g.	Pretreatment for 7 d, q.d.	Bcl-2↑; Bax↓, Cyto-c↓, Caspases-3/9↓, cleaved PARP-1↓, TUNEL-positive neurons↓	Wang T. et al., 2014
Rhizoma Pinelliae Pedatisectae	SD	Male	250–300	MCAO (2 h)/R (24 h)	5, 10, and 20 mg/kg, i.g.	Pretreatment for 7 d, b.i.d.	SOD↑, Bcl-2↑; Bax↓, MDA↓, TNF-α↓, IL-1 β↓, TUNEL-positive neurons↓	Ye et al., 2016
*Clinacanthus nutans* Lindau	Long-Evans	Male	——	MCAO (0.5 h)/R (24 h)	10–60 pg, icv; 24 mg/kg, i.p.	Single dose and 30 min after MCAO; pretreatment for 1 h or posttreatment for 3–24 h	PPAR-γ↑, C/EBPβ↑, 14-3-3ϵ↑, p-Bad↑, Bad↑, Bcl-2↑; cleaved Caspase-3↓, PARP↓	Wu J. et al., 2017
Spatholobi Caulis extract	SD	Male	240–260	MCAO (0.75 h)/R (7 d)	100 and 200 mg/kg, i.g.	Pretreatment for 3 d and posttreatment for 7 d, q.d.	BDNF↑, β-III-tubulin↑, ROS↓, GFAP↓, cleaved PARP↓, cleaved Caspases-3↓, p-p38↓, p-JNK↓, TUNEL-positive neurons↓	Park et al., 2018
Radix Scrophulariae aqueous extract	KunMing mice	Male	18–22	MCAO (2 h)/R (22 h)	2.4 g/kg, i.g.	Pretreatment for 7 d; q.d.	Bcl-2↑, SOD↑, MDA↓, NO↓, Bax↓, p-ERK1/2↓, p-P38↓	Meng et al., 2018

↑, upgrade; ↓, downgrade.

**Table 5:** The *in vitro* mechanism underlying the inhibition of MPTP opening-induced neuronal apoptosis by herbal medicine in the treatment of ischemic stroke.

Agents	Cell lines	Model	Dose	Time periods	Mechanisms	References
ABP	PHN of embryonic, 18-d SD rats	OGD (NMDA insult, 0.5 h)/R (24 h)	1 µg/ml	Pretreatment for 12 h and during OGD/R period	MMP↑; Bax↓, Caspase-3↓, ROS↓	Shen et al., 2010
GLP	PCN of neonatal SD rats (<24 h)	OGD (5% CO_2_ and 95% N_2,_ 2 h)/R (24 h)	0.1, 1.0, and 10.0 μg/ml	Pretreatment for 0.5 h and during OGD/R period	Bcl-2↑; cleaved Caspases-3/8/9↓, Bax↓	Zhou et al., 2010
Astragalosides	PC12	OGD (5% CO_2_ and 95% N_2,_ 5 h)/R (24 h)	1, 100, and 200 g/ml	During reperfusion period	MMP↑, p-p38/p38↑,; fragmented DNA↓, LDH↓, Caspase-3/9/12↓, cleaved Caspases-3/9↓, ROS↓, LC3–11↓, Bip↓, neuronal apoptosis under flow cytometry↓,	Chiu et al., 2014
*Clinacanthus nutans* Lindau	PCN of embryonic, 15.5-d Balb/c mouse	OGD (0.02–0.1% O_2_, 5% CO_2_, 10% H_2_, and 85% N_2_, 0.5 h)/R (4–24 h)	6.25 μg/ml	Pretreatment for 1 h and during OGD/R period	14-3-3ϵ↑, C/EBPβ↑, PPAR-γ↑, p-Bad↑, Bcl-2↑, MMP↑; cleaved Caspase-3↓, PARP-1↓	Wu J. et al., 2017
LBP	PHN of neonatal SD rats (<24 h)	OGD (5% CO_2_ and 95% N_2,_ 2 h)/R (24 h)	10, 20, and 40 mg/L	During reperfusion period	MMP↑, IκB-α↑, LDH↓, ROS↓, Ca^2^^+^↓, IL-6↓, TLR4↓, NF-κB↓, Hoechst 33342 positive neurons↓, TUNEL-positive cells↓	Zhao et al., 2017b
Spatholobi Caulis extract	SH-SY5Y	A-24 h etoposide insult	25 and 50 μg/ml	Pretreatment for 6 h and co-culture with etoposide for 24 h	MMP↑; cleaved PARP↓, p-p53↓, cleaved Caspase-3↓, Caspase-3/7↓, p-JNK/JNK↓, p-p38 /p38↓, TUNEL-positive neurons↓, neuronal apoptosis under flow cytometry↓	Park et al., 2018
Radix Scrophulariae aqueous extract	PC12	OGD (5% CO_2_ and 95% N_2,_ 2 h)/R (24 h)	6.25, 12.50, 25.00, and 50.00 µg/ml	Pretreatment for 24 h	Bcl-2↑, SOD↑, GSH-Px↑, CAT↑, MMP↑; LDH↓, MDA↓, NO↓, Bax↓	Meng et al., 2018
CDP	PC12	OGD (5% CO_2_ and 95% N_2_, 4 h)/R (24 h)	0.05, 0.50, and 5.00 μg/ml	During reperfusion period	CAT↑, GSH-Px↑, T-AOC↑, MMP↑, DJ-1↑; ROS↓, LDH↓, MDA↓, Ca^2+^↓, neuronal apoptosis under flow cytometry↓, Hoechst33342 positive neurons↓	Liu et al., 2018
*Scutellaria barbata* D. Don extract	PC12	OGD (1% O_2,_ 5% CO_2_, and 94% N_2_, 6 h)/R (18 h)	0.1–0.8 mg/ml	Pretreatment for 12–48 h	p-PI3K/ PI3K↑, p-AKT/AKT↑, p-PI3K↑, p-AKT↑, Nrf2↑, SOD↑, GSH↑, MMP↑, Ki67 positive cells↑,Cycin D1↑, Cyclin E↑; MDA↓, Bax↓, cleaved Caspase-3↓, Bid↓, neuronal apoptosis under flow cytometry↓	Wang et al., 2019e
*Aglaia odorata* Lour. extract	PC12	OGD (5% CO_2_ and 95% N_2,_ 4 h)/R (24 h)	5, 10, and 50 ng/ml	During OGD/R period	MMP↑, Caspase-3↑, PARP↑, Bcl-2↑; LDH↓, cleaved PARP↓, cleaved Caspase-3/9↓, Bax↓, p53↓, Puma↓, mtROS↓, AO/EB and Hoechst 33258 positive neurons↓	Wang et al., 2020

↑, upgrade; ↓, downgrade.

### Monomers and Molecular Mechanisms in Regulating MPTP Openness of Ischemic Stroke

**Figure 3:**
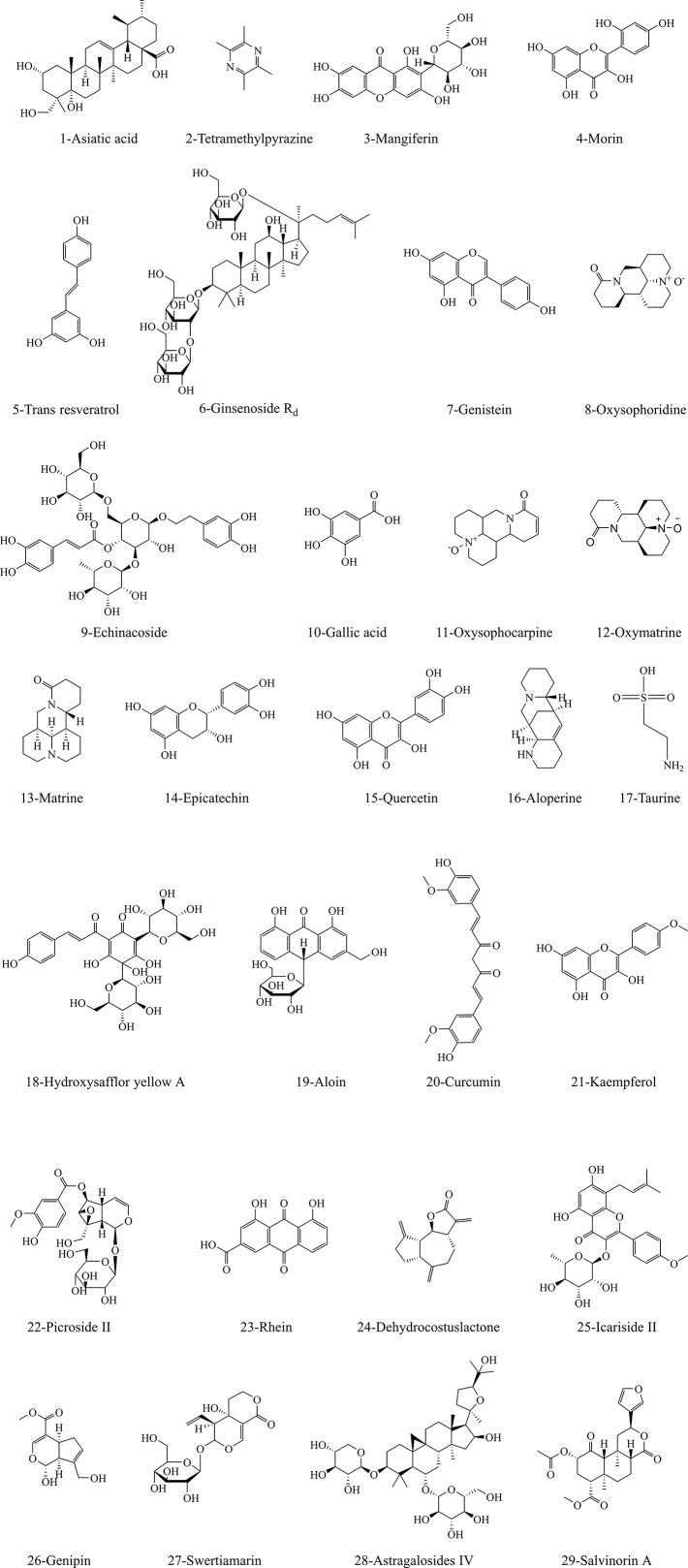
The structural information of underlying compounds for regulation of MPTP opening to inhibit apoptosis in ischemic neurons. The structural formulae of 29 monomer compounds are shown in the figure.

As research continues, massive active ingredients for treating stroke have been identified from herbal medicines. According to literature reports, we summarized 29 monomer compounds that may target to inhibit mitochondrial MPTP overopening-induced neuronal apoptosis, including alkaloids, flavonoids, terpenoids, and phenolic acids. **Figure 3** shows the structure information of these potential compounds. **Tables 6** and **7** list the specific brain protective mechanisms of monomer compounds against ischemia-induced neuronal apoptosis. Notably, some of these compounds have been shown to regulate MPTP to improve ischemic stroke. The anti-oxidant and anti-inflammatory effects of hydroxy safflor yellow A (HSYA) and carboxyatractyloside could help to inhibit ischemia-induced MPTP opening and play a protective role against cerebral ischemia (Ramagiri and Taliyan, 2016). The anti-hypoxic effect of kaempferol was related to inhibition of mitochondrial fission, maintenance of mitochondrial integrity and function, and therefore repressing MPTP opening-induced apoptosis (Wu B. et al., 2017). *In vivo* and *in vitro* experiments showed that the protective effect of gallic acid (GA) on cerebral ischemia against apoptosis might be related to inhibition of oxidative stress response, Ca^2+^ and ROS overproduction-evoked MPTP opening, and the transfer of mitochondrial Cyto-c to the cytoplasm, and thus increasing mitochondrial ATP supply and MMP (Sun et al., 2014). The authors further illuminated that GA could inhibit MPTP-induced apoptosis by regulating ERK-CypD axis, which may make GA a natural MPTP opening inhibitor for treating ischemic stroke (Sun et al., 2017). Earlier studies have reported that the anti-oxidative stress and apoptotic properties of trans resveratrol (Agrawal et al., 2011) and resveratrol (Narayanan et al., 2015) may lead to a protective effect against ischemia. Subsequently, studies confirmed that OGD/R induced bEND3 cerebrovascular endothelial cell edema was associated with monocyte chemoattractant protein and intracellular Ca^2+^ overload, while resveratrol could maintain mitochondrial MMP by inhibiting ROS and elevated Ca^2+^ ions, thus improving hypoxic brain edema (Panickar et al., 2015). Excitingly, recent study has further demonstrated that the anti-anoxic brain protection of preadministrated resveratrol may be related to consolidating mitochondrial tolerance to anoxia and increasing VDAC level and energy synthesis (Khoury et al., 2019). However, on the contrary, picroside II could interdict release of pro-apoptotic factor Endo G into the nucleus driven by MPTP opening, ROS production and VDAC1 protein expression (Li S. et al., 2018). Therefore, it is worth further focus on VDAC, one of the main components of MPTP, as an interesting target for stroke treatment.

**Table 6:** The *in vivo* mechanism underlying the inhibition of MPTP opening-induced neuronal apoptosis by monomeric compounds in the treatment of ischemic stroke.

Agents	Objects	Gender	Weight (g)	Animal model	Dose	Time periods	Mechanisms	References
Asiatic acid	C57BL/6	male	22–27	pMCAO	30, 75, and 165 mg/kg, i.g.	1 h before and 3, 10, and 20 h after pMCAO	Cyto-c↓, BBB permeability (IgG)↓	Krishnamurthy et al., 2009
Ginsenoside R_d_	SD	male	270–320	MCAO	50 mg/kg, i.p.	30 min before MCAO	MMP↑, aconitase↑, mitochondrial complexes I-IV↑; ROS↓, lactate/pyruvate ratio↓, cleaved Caspase-3, Cyto-c↓, AIF↓	Ye et al., 2011b
Genistein	C57/BL6J	male	24–28	MCAO (1 h)/R (24 h)	2.5, 5, and 10 mg/kg, i.g.	Pretreatment once daily for 2 w	SOD↑, GSH-Px↑, mitochondrial Cyto-c↑; MDA↓, mtROS↓, cytosolic Cyto-c↓, Caspase-3↓, TUNEL-positive neurons↓, p-NF-κB p65 subunit↓, p-IκBα↓	Qian et al., 2012
Oxysophoridine	ICR	male	20–25	MCAO (2 h)/R (24 h)	62.5, 125, and 250 mg/kg, i.p.	Pretreatment once daily for 1 w	SOD↑, GSH-Px↑, Bcl-2↑; MDA↓, Caspase-3↓, Bax↓, TUNEL-positive neurons↓	Wang et al., 2013
Echinacoside	SD	both	12–17	Permanent ligation of the left CCA plus low oxygen atmosphere (8% O_2_, 92% N_2_) for 2.5 h	40, 80, and 160 mg/kg, i.p.	Every 12 h after operation, a total of 4 times	SOD↑, GSH-Px↑, CAT↑, T-AOC↑, Bcl-2/Bax↑; MDA↓, Caspase-3↓, TUNEL-positive neurons↓	Wei W. et al., 2019
Gallic acid	SD	male	250–300	MCAO (2 h)/R (24 h)	25 and 50 mg/kg, i.v.	20 min before MCAO	MMP↑, mitochondrial Cyto-c↑; MDA↓, ROS↓, cytosolic Cyto-c↓, TUNEL-positive neurons↓	Sun et al., 2014
	SD	male	250–300	MCAO	50 mg/kg		binding capacity of CypD and ANT-1↓, MPTP openinng↓, p-ERK↓, Cyto-c↓, cleaved Caspase-3/8/9↓	Sun et al., 2017
Oxymatrine	SD	both	——	Permanent ligation of the left CCA plus low oxygen atmosphere (8% O_2_, 92% N_2_) for 2.5 h	30, 60, and 120 mg/kg, i.p.	Every 12 h after operation, a total of 2 times	SOD↑, GSH-Px↑, CAT↑, T-AOC↑, Bcl-2/Bax↑, MDA↓, Caspase-3↓, neuronal apoptosis under flow cytometry↓	Zhao et al., 2015a
Matrine	ICR	male	20–25	MCAO (2 h)/R (24 h)	7.5, 15, and 30 mg/kg, i.p.	Pretreatment once daily for 1 w	SOD↑, GSH-Px↑, CAT↑, T-AOC↑, Bcl-2/Bax↑, MDA↓, Caspase-3↓, neuronal apoptosis under flow cytometry↓	Zhao et al., 2015b
Taurine	SD	both	——	Permanent ligation of the left CCA plus low oxygen atmosphere (8% O_2_, 92% N_2_) for 2 h	30, 60, and 120 mg/kg, i.p.	Every 12 h after operation, a total of 2 times	SOD↑, GSH-Px↑, T-AOC↑, Bcl-2/Bax↑, ATP↑; LA↓; MPO↓, MDA↓, AIF↓, Cyto-c↓	Zhu et al., 2016
HSYA	Wistar	male	220–250	MCAO (1 h)/R (24 h)	8 mg/kg, i.v.	After reperfusion	GSH↑, CAT↑; MDA↓, TNF-α↓, MPTP opening↓	Ramagiri and Taliyan, 2016
Curcumin	Wistar	male	180–200	MCAO/R	25 mg/kg, i.p.		Bcl-↑, Sirt1↑, MMP ↑; p53↓, Bax↓, IL-6↓, TNF-α↓, ROS↓	Zhang et al., 2017
Picroside II	Wistar	male	240–260	MCAO (2 h)/R (24 h)	20 mg/kg, i.p.	15 min before MCAO/R.	VDAC1↓, cytoplasmic and nuclear EndoG↓, ROS↓, MPTP opening↓, TUNEL-positive neurons↓	Li S. et al., 2018
Rhein	SD	male	260–300	MCAO (2 h)/R (72 h)	25, 50, and 100 mg/kg, i.g.	3 days following MCAO/R	SOD↑, GSH-Px↑, CAT↑, Bcl-2/Bax ratio↑; MDA↓, Caspase-3/9↓, cleaved Caspase-3↓	Zhao et al., 2018b
Genipin	C57BL/6	male	25–30	MCAO (1 h)/R (24 h)	50 mg/kg, i.g.	Pretreatment once daily for 3 d	ATP↑, SOD↑, GSH↑; UCP2↓, SIRT3↓, NAD^+^/NADH↓, LDH↓, cleaved Caspase-3↓, TUNEL-positive neurons↓	Zhao B. et al., 2019
Swertiamain	ICR	male	20–25	MCAO (2 h)/R (24 h)	25, 100, and 400 mg/kg, i.p.	Pretreatment once daily for 1 w	Bcl-2/Bax↑, SOD↑, GSH-Px↑, CAT↑, GSH↑, nulcear Nrf2↑, HO-1↑, NQO1↑; MDA↓, Keap1↓, cytoplasmic Nrf2↓, TUNEL-positive neurons↓	Wang et al., 2019b

↑, upgrade; ↓, downgrade.

**Table 7:** The *in vitro* mechanism underlying the inhibition of MPTP opening-induced neuronal apoptosis by monomeric compounds in the treatment of ischemic stroke.

Agents	Cell lines	Model	Dose	Time periods	Mechanisms	References
Asiatic acid	HT-22	OGD (5 h)/R (24 h)	1 and 10 µg/ml	Posttreatment for 24 h	MMP↑, Cyto-c↓	Krishnamurthy et al., 2009
Tetrahydroxystilbene glucoside	PCN of neonatal SD rats	OGD (5% CO_2_ and 95% N_2_, 2 h)/R (24 h)	25 µM	Pretreatment for 24 h	MMP↑, SIRT1↑, Bcl-2/Bax↑; LDH↓, ROS↓, p-JNK↓, iNOS↓, nuclear p65↓, Ca^2+^↓, Hoechst 33258 positive staining↓	Wang et al., 2009
Mangiferin and Morin	PCN of embryonic SD rats	50 μM glutamate plus 10 μM glycine	1–10^4^ nM	During and after glutamate exposure	SOD↑, CAT↑, p-Akt↑, cytoplasmic p65↑, MMP↑; Calpain↓, p-Erk1/2↓, nuclear p65↓, AIF↓, Bax↓, ROS↓	Campos-Esparza et al., 2009
Trans resveratrol	PC12	OGD (5% CO_2_, 94% N_2_, and 1% O_2_, 6 h)/R (24 h)	5, 10, and 25 μM	24 h before/post OGD	Bcl-2↑, GSH↑; Bax↓, HIF-1α↓, Caspase-3↓, ROS↓, LPO↓	Agrawal et al., 2011
Oxysophoridine	PHN of neonatal SD rats	OGD (2 h)/R (24 h)	5, 20, and 80 μM	OGD (2 h)/R (24 h)	Bcl-2/Bax↑; Caspase-3/8/9↓, Cyto-c↓, Hoechst-33342 fluorescence intensity↓ SOD↑, CAT↑, GSH-Px↑, MMP↑; NOS↓, glutamate↓, Ca^2+^↓, MDA↓, NO↓	Chen et al., 2013; Zhao et al., 2013
Gallic acid	SH-SY5Y	Hypoxia (Na_2_S_2_O_4_, 2 h)/R (2 h)	0.1, 1, and 10 μM	Pretreatment for 24 h	MMP↑, ATP↑, oxygen consumption↑; MDA↓, intracellular ROS↓, mtROS↓, MPTP opening↓	Sun et al., 2014
	SH-SY5Y	/	0.1, 1, and 10 mM	24 h before H_2_O_2_-induced MPTP opening	binding capacity of CypD and ANT-1↓, MPTP openinng↓, p-ERK↓, Cyto-c↓, cleaved Caspase-3/8/9↓	Sun et al., 2017
Oxysophocarpine	PHN of neonatal SD rats	OGD (5% CO_2_, and 95% N_2_, 2 h)/R (24 h)	1, 2, and 5 μmol/L	During reperfusion period	MMP↑; LDH↓, Ca^2+^↓, Caspase-3/12↓	Zhu et al., 2014
Epicatechin and Quercetin	PCN of embryonic CD1 mice	OGD (5% CO_2_, 5% H_2_, and 90% N_2_, 5 min)/R (1.5 h)	0.1–10 μM	Pretreatment for 24 h	OCRs↑, p-Akt/Akt↑, p-CREB/CREB↑, Bcl-2↑, PGC-1a↑, MT-ND2 (complex I)↑, MT-ATP6 (complex V)↑, MMP↑; Ca^2+^↓, NOS↓	Nichols et al., 2015
Aloperine	PHN of neonatal SD rats	OGD (5% CO_2_, and 95% N_2_, 2 h)/R (24 h)	25, 50, and 100 mg/L	During reperfusion period	CAT↑, SOD↑, GSH-Px↑, T-AOC↑, MMP↑; LDH↓, Ca^2+^↓, MDA↓, ROS↓, Hoechst 33342 positive staining↓	Ma et al., 2015
Aloin	PC12	OGD (5% CO_2_, and 95% N_2_, 4 h)/R (24 h)	10, 20, and 40 μg/ml	During OGD/R period	MMP↑, Bcl-2↑, SOD↑; LDH↓, MDA↓, ROS↓, Ca^2+^↓, Bax↓, Caspase-3↓, Hoechst 33342 positive staining↓, apoptosis under flow cytometry↓	Chang R. et al., 2016
Kaempferol	PCN of 17-d embryonic rats	OGD (2 h)	10 μM	Before OGD	OCRs↑, p-Akt/Akt↑, MMP↑, p-Drp1/Drp1↑, ATG5↑, ATP↑, HK-II↑, LC3 II/I ratios↑, mitochondrial Cyto-c/cytosolic Cyto-c↑; ROS↓, Ca^2+^↓, SDH↓, apoptosis under flow cytometry↓, MPTP openinng↓	Wu B. et al., 2017
Dehydrocostuslactone	hippocampal slices of SD rats	OGD (5% CO_2_, and 95% N_2_, 0.5 h)/R (1 h)	1, 5, and 10 µM	During OGD/R period	LC3 II/I ratios↑, Bcl-2↑; LDH↓, Bax↓, Cyto-c↓, Apaf-1↓, Caspase-3/7/9↓, p62↓	Zhao et al., 2018a
Icariside II	PC12	OGD (5% CO_2_, and 95% N_2_, 2 h)/R (24 h)	12.5, 25, and 50 μM	Posttreatment for 24 h	nuclear Nrf2↑, NQO-1↑, HO-1↑, Bcl-2/Bax↑, SIRT3↑, IDH2↑_,_ MMP↑; LDH↓, ROS↓, cytoplasmic Nrf2↓, Keap1↓, cleaved Caspase-3↓, TUNEL-positive neurons↓	Feng et al., 2018
Astragaloside IV	PCN of 18-d embryonic SD rats	OGD (1% O_2_, 5% CO_2_, 3 h)/R (24 h)	6.25, 12.5, and 25 μmol/L	During OGD/R period	p-PKA/PKA and p-CREB/CREB↑, ATP↑, MMP↑; LDH↓, cleaved Caspase-3↓, ROS↓	Xue et al., 2019
Oxymatrine	PHN of newborn SD rats	OGD (5% CO_2_ and 95% N_2_, 2 h)/R (24 h)	0.2, 1, and 5 µg/ml	During reperfusion period	MCL-1↑, Bcl-2↑, p-Akt↑, p-PI3K↑, p-GSK3β↑, MMP↑; LDH↓, Ca^2+^↓, Caspase-3↓, NR2B↓ (NMDAR1), TUNEL-positive neurons↓, neuronal apoptosis under flow cytometry↓	Liu Y. et al., 2019
Salvinorin A	HBMECs	OGD (5% CO_2_ and 95% N_2_, 6 h)/R (24 h)	5 uM	During reperfusion period	p-AMPK↑, Mfn2↑, ATP↑, MMP↑; ROS↓, Ca^2+^↓, apoptosis under flow cytometry↓	Dong et al., 2019

↑, upgrade; ↓, downgrade.

*In vivo* and *in vitro* studies have shown that oxysophoridine could regulate Bcl-2 family members, and thereby counteracting mitochondria-mediated apoptosis. Meanwhile, it was possible to suppress Ca^2+^ overload of neurons and maintain mitochondrial MMP by anti-oxidative stress and inhibiting the toxicity of neuronal excitatory amino acids (Chen et al., 2013; Wang et al., 2013; Zhao et al., 2013). Oxysophocarpine could also limit hypoxia-induced neuronal apoptosis by inhibiting Ca^2+^ and increasing MMP (Zhu et al., 2014). Further studies have shown that the inhibitory effect of apoptosis was related to anti-inflammatory and down-regulation of MAPK signaling pathway (Zhao et al., 2017a). Similarly, aloperine (Ma et al., 2015), matrine, and oxymatrine (Zhao et al., 2015a; Zhao et al., 2015b; Liu Y. et al., 2019) may have the same protective effect against ischemic neuron apoptosis. As a reversible selective inhibitor of true cholinesterase, huperzine A has been shown to inhibit mitochondrial complexes I–IV, a-ketoglutarate dehydrogenase, and MMP decline after ischemia, which helps to eliminating excessive ROS and Ca^2+^ (Zheng et al., 2008). Considering the short *in vivo* half-life of tetramethylpyrazine, a novel compound containing tetramethylpyrazine and carnitine structures was synthesized. Further *in vivo* and *in vitro* results also confirmed that its anti-hypoxic brain protective effect was related to anti-oxidative stress and anti-inflammatory, ultimately maintaining the morphology and function of neurons and inhibiting neuronal apoptosis (Wang et al., 2017). Of course, there are other natural compounds that antagonize ischemia-infuriated morphological and functional disorders of brain mitochondria by regulating oxidative stress signals such as leonurine (Loh et al., 2010) and neferine (Wu C. et al., 2019).

Flavonoids resisting oxidative stress may drive the recovery of ischemia attacked neuron mitochondrial function, evidenced by increased mitochondrial biosynthesis and respiration, dampened Ca^2+^ production, and mitochondria edema, such as icariside II (Feng et al., 2018), as well quercetin and epicatechin in flavonols (Nichols et al., 2015). As an Nrf2 activator, mangiferin inhibited the nuclear translocation of two subunits of NF-κB, p65 and p50, and the superior antioxidant properties of mangiferin and morin inhibited Ca^2+^ overload and improved mitochondrial MMP, thus counteracting the lethal post-ischemic neuronal excitatory toxic damage and cascade apoptosis (Campos-Esparza et al., 2009). Other reports suggested that the protective effects of genistein (Qian et al., 2012), isorhamnetin (Li et al., 2016), and vitexin (Cui et al., 2019) against ischemia may involve both inflammation and inhibition of neuronal apoptosis. Most terpenoids also have antioxidant properties similar to those of alkaloids and flavonoids, which helped maintain mitochondrial morphology and respiratory function as well as ischemia-induced neuronal apoptosis, such as bilobalide (Schwarzkopf et al., 2013) and Swertiamain (Wang et al., 2019b). Studies have shown that the treatment time window of asiatic acid can be maintained for at least 12 h, which is related to the improvement of MMP and the inhibition of mitochondrial Cyto-c release (Krishnamurthy et al., 2009). The balanced redox effect of ginsenoside Rd may contribute to the improvement of cerebral injury symptoms (Ye et al., 2011a). Further evidence showed that Rd could improve mitochondrial respiratory function and increase ATP production by reducing ROS production, thereby maintaining MMP and inhibiting neuronal apoptosis (Ye et al., 2011b), which was similar to dehydrocostuslactone’s protection of rat hippocampal slices from OGD/R-induced damage (Zhao et al., 2018a). Astragalosides IV may maintain mitochondrial function and inhibit OGD/R-induced cortical neuronal apoptosis by regulating PKA/CREB signaling pathway (Xue et al., 2019). *In vivo* and *in vitro* evidence suggested that Salvinorin A played an anti-apoptotic and anti-hypoxia protective role in brain involving the reduction of ROS and Ca^2+^ production in cerebrovascular endothelial cells, the activation of AMPK/Mfn2 signaling pathway, and ultimately maintenance of mitochondrial morphology and MMP (Dong et al., 2019). As an excellent natural biological cross-linking agent and a specific inhibitor of mitochondrial uncoupling protein 2 (UCP2), *in vivo* studies have shown that genipin could improve mitochondrial energy metabolism by inhibiting UCP2-SIRT3 signaling pathway to mitigate oxidative stress injury and neuronal apoptosis after hypoxic brain injury (Zhao B. et al., 2019).

**Figure 4:**
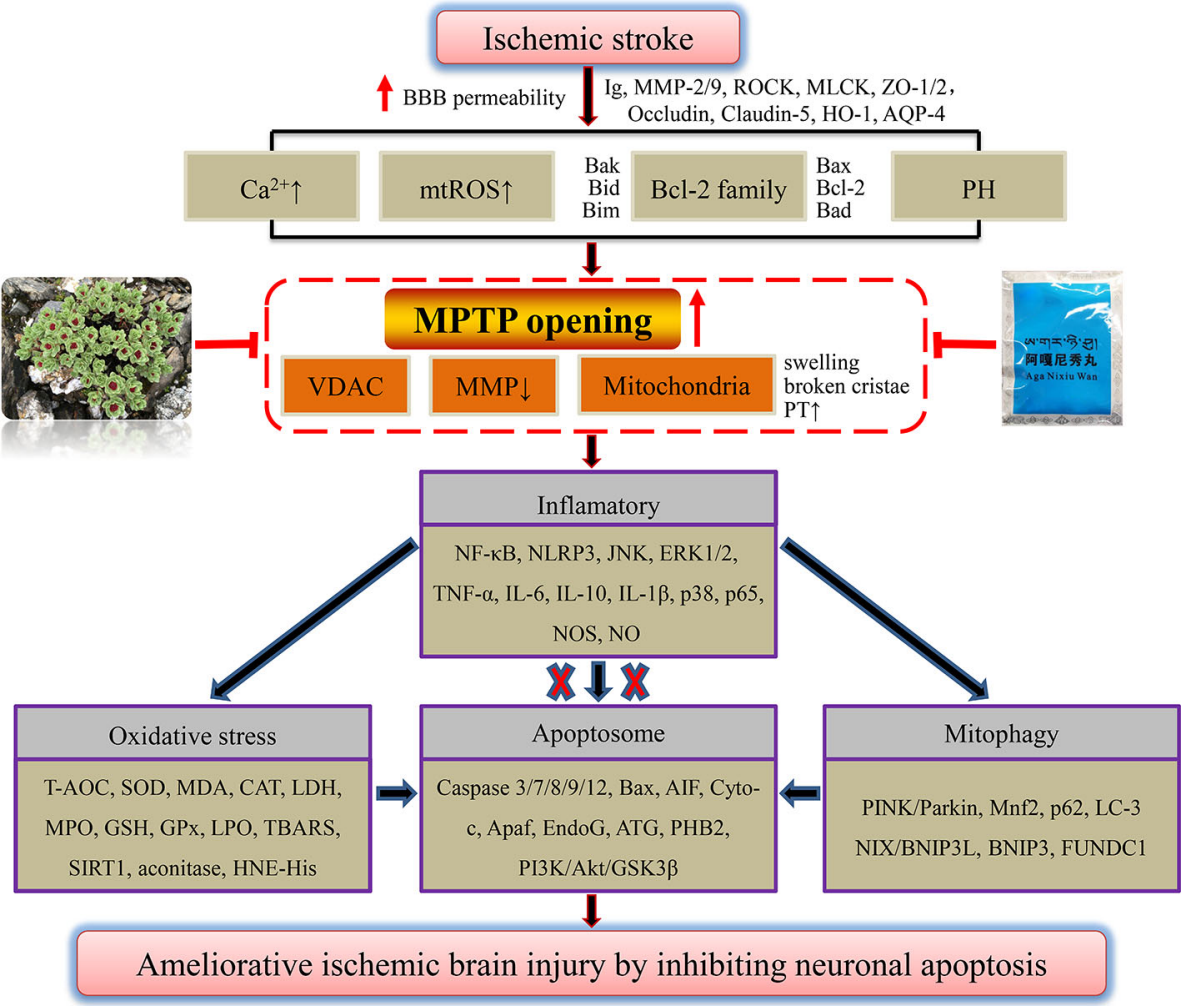
A panoramic view of natural products inhibiting MPTP opening-induced neuronal apoptosis in the treatment of ischemic stroke. Any adverse stimuli after ischemic stroke could favor MPTP opening. However, natural products that inhibit MPTP opening could further prevent neuronal inflammation after ischemia, oxidative stress injury, and mitophagy, and finally repress ischemic neuron apoptosis.

Other compounds such as taurine (Zhu et al., 2016) and echinacoside (Wei W. et al., 2019) could also regulate Bcl-2 family members through antioxidant stress, and inhibit mitochondrial apoptosis to improve hypoxic brain injury. Ischemic brain protection against neuronal apoptosis of phenolic acid compounds tetrahydroxystilbene glucoside (Wang et al., 2009), vanillin (Lan et al., 2019), curcumin (Zhang et al., 2017), and apocynin (Connell et al., 2012) may also further involved in the mechanism of anti-inflammatory, such as regulating the NF-κB and JNK, or targeting SIRT1. The antioxidant activity of quinones shikonin (Wang et al., 2010) and aloin (Chang R. et al., 2016), with a similar anti-cerebral ischemia action of rhein in our previous study (Zhao et al., 2018b), as well as phenylpropanoid compounds cinnamtannin D1 and trans-cinnamaldehyde (Panickar et al., 2015; Qi et al., 2016) from cinnamon might reduce the accumulation of Ca^2+^ and ROS, thus improving MMP to exert anti-ischemic neuron apoptosis. Through the above analysis of officially authorized drugs for the treatment of ischemic stroke, ethnic drug prescription, herbs, and monomer components, we found that most of them have the effect of anti-oxidative stress. The inhibition of overloaded Ca^2+^ and overproduced mtROS is the premise of drugs to reverse the decline of MMP after ischemia, improve mitochondrial respiratory function, and maintain the ATP supply of neurons. Although apoptosis might be the ultimate destination of neurons after ischemic stroke, we are pleasantly surprised to find that many adverse factors after ischemia might drive mitochondrial MPTP overopening. Meanwhile, we have previously discussed some potential proteins or oligomers that may be involved in regulating MPTP opening after cellular hypoxia, such as Bcl-2, Bax, Bcl-xL, and oligomer Bax/Bak of the Bcl-2 family. Through reviewing literatures, we also found that the above natural products could directly or indirectly inhibit MPTP overopening after ischemia. Furthermore, increased OMM permeability and collapsed mitochondrial membrane structures are inhibited. Ultimately, the integrity of the mitochondrial membrane and MMP are rescued, thus inhibiting the vicious cycle of excessive Ca^2+^ and mtROS production. As seen from the end results, caspase-dependent apoptosis triggered by the release of mitochondrial contents such as Cyto-c and AIF was blocked. Collectively, we have reason to believe that mitochondrial MPTP may be a potential target of natural products to inhibit neuronal apoptosis in treatment of ischemic stroke. Among the mechanisms, there may also be inflammation and oxidative stress signaling involved in MPTP opening and apoptosis. We summarized the mechanisms by which ethnic drugs may regulate MPTP to inhibit apoptosis of ischemic neurons, as shown in **Figure 4**. Among them, the mechanisms that have not been reported and elucidated still need to be further probed.

## Conclusion and Future Prospects

The literatures on targeted improvement of mitochondrial MPTP by ethnic medicine were reviewed systematically and purposefully. We were ecstatic to accept the trend that balanced mitochondrial MPTP was becoming a novel strategy for drug treatment of stroke (Briston et al., 2019). First, we identified that the process of stroke was associated with an abnormal over-opening of mitochondrial MPTP. Any factors that induced insufficient blood supply to the brain may lead to robust ROS, unbalanced intracellular Ca^2+^ homeostasis, decrease MMP, inflammation, and ERS. These detrimental events were doomed to be fatal to mitochondria and initiate changes in the three-dimensional conformation of mitochondrial MPTP, which would in turn aggravate the production of mitochondrial ROS, mitochondrial edema, the booming cytoplasmic Ca^2+^, the decline of MMP, and the reduction of ATP synthesis. To sum up, all these adverse biological events that caused the loss of the function of mitochondrial bilayer barrier would inevitably disrupt the material transfer between mitochondrial matrix and cytoplasm. Consequently, the activated mitochondrial dependent apoptosis was triggered according to an inherent set of biological procedures. And this process was regularly and strictly executed by mitochondria-emitted apoptosis signal, and delivered step by step. For instance, ischemia-induced MPTP opening leaded to the translocation of Cyto-c from mitochondrial matrix into cytoplasm, and binding with Apaf-1 and Caspase-9 to form apoptosome, thereby activating caspase-dependent programmed cell death pathways in ischemic/anoxic neurons. Secondly, we found piece by piece that ethnic drug prescriptions, herbal medicine, and monomer components could participate in regulation of excessive MPTP opening induced-ischemic neuronal apoptosis from different perspectives. We therefore concluded that mitochondrial MPTP, a very considerably intermediate link in apoptosis signaling, might be a novel target for natural products in treatment of stroke.

However, by weighing the pros and cons, the following aspects should be worthy to further optimization considering the anti-apoptotic brain protection effect of ethnic drugs through regulation of mitochondrial MPTP. First, the complexity and uncertainty of active ingredients penetrating blood brain barrier (BBB). Current methods for identifying active ingredients included high performance liquid chromatography (HPLC), mass spectrum, gas chromatography-mass spectrometer (GC-MS), or liquid chromatograph-mass spectrometer (LC-MS). However, the key problem lay in the selection and preprocessing of samples for content determination: the original herbs or prescription extracted by simple decoction, ultrasound or different proportions of organic reagents, animal serum or brain tissue homogenate after administration. Any test based on those ideas would simply identify specific monomer compounds contained in certain prescriptions or extracts. However, the premise of drug efficacy was to achieve a certain concentration in target organs or tissues such as specific brain regions to stimulate the transmission of anti-apoptotic protective signals. Slightly regretfully, the qualitative or quantitative identification methods mentioned above cannot completely represent the concentration of drug enrichment in cerebral ischemic regions. For this existing and confronting problems, we proposed that a microdialysis device coupled HPLC/MS would be a potential platform for screening active ingredients (Reyes-Garcés et al., 2019) or changeable pH value of brain microenvironment (Su and Ho, 2019). Moreover, distribution concentrations of different small molecule drugs targeting distinct brain regions could be dynamically presented in real time and *in vivo* by an integrated platform of high resolution laser confocal microimaging coupled with brain MS imaging (He et al., 2019; Liu C. et al., 2019). Finally, a multi-dimensional image of drug distribution in brain tissue was visually and stereoscopically constructed. Second, the rationality of *in vivo* and *in vitro* simulation of clinical stroke model in light of the complexity of BBB tissue structure (Sweeney et al., 2019). Currently, diverse *in vivo* stroke models for cerebral ischemia, or *in vitro* OGD/R-induced hippocampal slices or different neuron injury models, which were widely accepted and acquiescent, cannot reproduce the scene of changes in brain tissue structure and the specific molecular-mediated damage mechanisms yet. Therefore, the above existing stroke models needed to be further discussed. However, it was encouraging to note that our research group had successfully established *in vitro* co-culture models of cerebrovascular endothelial cell, astrocytes, and pericytes to simulate BBB (the data have not yet been published), referring to the organ-like models of multiple neurons co-culture or BBB previously reported (Bergmann et al., 2018). Of course, through establishment of *in vitro* neurovascular unit (NVU), we also strived to achieve real-time and rapid evaluation of natural small molecule compounds passing BBB, and to screen the quality markers of ethnic drugs and functional protein targets on a coupled microfluidic chip-mass spectrometry (MC-MS) platform (Wang et al., 2019d).

**Figure 5:**
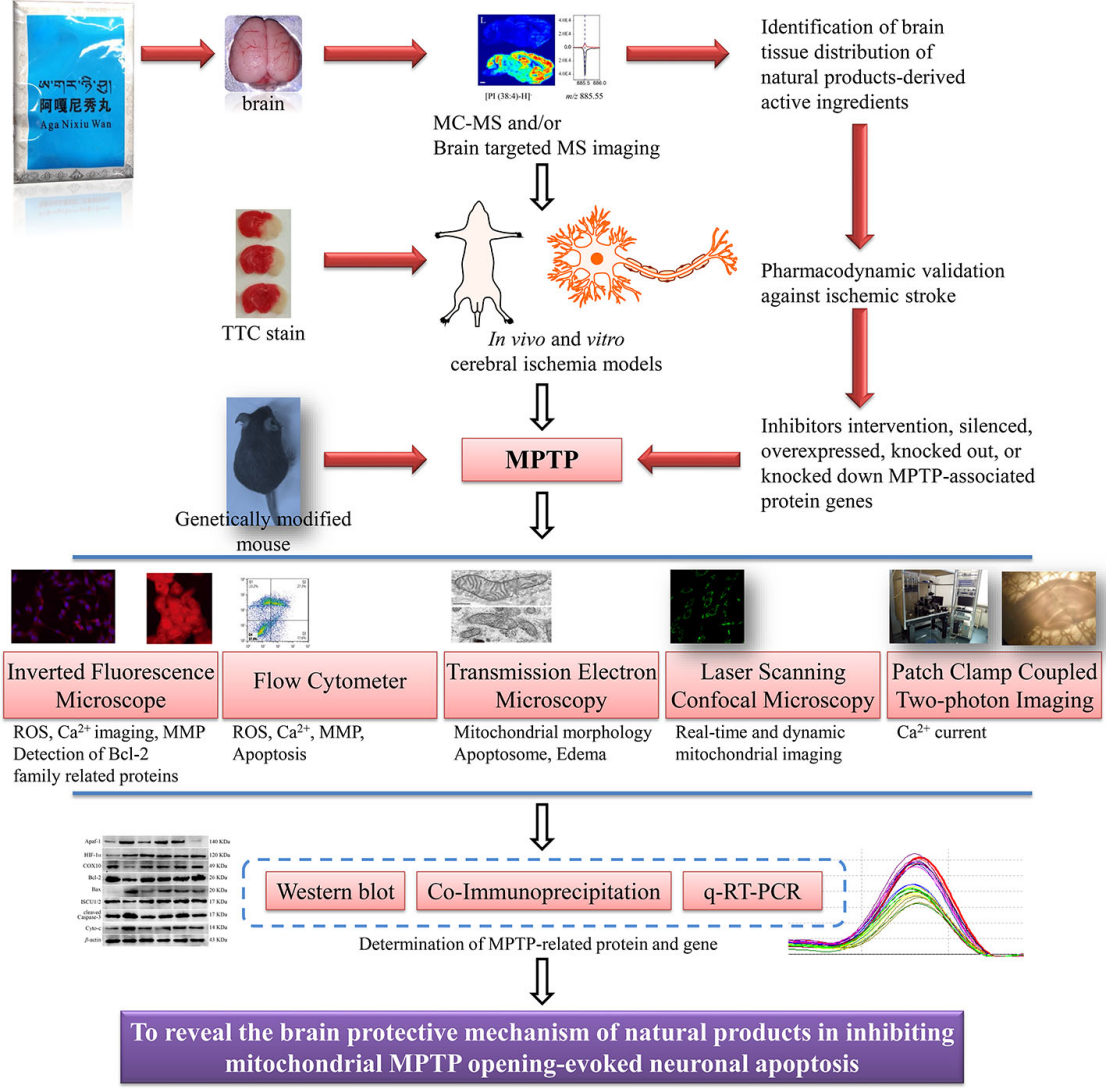
Conceptual flowchart of combined multiple techniques for MPTP regulation by natural products on apoptosis of ischemic neurons. Mitochondrial MPTP is a novel target for the treatment of ischemic stroke. Determination of the distribution of natural products in distinct brain regions, reasonable *in vivo* and *in vitro* stroke models, and advanced MPTP imaging technologies will be conducive to the development of ethnic drugs targeting MPTP.

Third, the mechanisms of small molecular compounds acting on mitochondrial MPTP to inhibit apoptosis after ischemic stroke were unsophisticated. According to what we have learned, the conventional means demonstrating the interrelationship between drugs and MPTP were limited to the following. After intervention with MPTP inhibitors or agonists, conventional western blot, immunohistochemistry/fluorescence (Bonora et al., 2016), flow cytometry, and qRT-PCR were employed to evaluate the effect of drugs on changes in protein and gene expression that made up MPTP, such as VDAC and ANT. In addition, gene editing such as plasmids or viruses transfection of target gene vectors to overexpress or silence the target gene, or to completely knock out or down the target gene and observe the effect of drugs on MPTP were also some popular molecular biology methods. Certain proteins or protein complexes such as Bax/Bak dimerization, mtROS, oxidative stress, and inflammatory factors could regulate MPTP opening-induced cell apoptosis, thus providing indirect evidence for drug regulation of MPTP. The more intuitive evidence might be to detect some of triggering hallmarks after MPTP opening, such as mitochondrial swelling, decreased MMP and ATP production, and detection of fluorescent labeled cytoplasmic Ca^2+^ surge. However, none of the above methods could provide direct evidence of drug-MPTP-apoptosis. That is, it cannot be visualized that drugs confined MPTP opening, and thus inhibiting cell apoptosis. The deficiencies of the above mechanisms investigation included the limited understanding of MPTP and the limitations of current molecular imaging technologies. Therefore, more efforts were needed to explore the molecular basis and regulatory mechanism of MPTP. We also had reason to believe that the laser confocal high intentionality live cell real-time imaging and analysis system would be a robust alternative for probing drug targeted regulation of MPTP. Moreover, patch-clamp combined with two-photon living cell imaging technology also had potential prospects for detection of prophylaxis and treatment of ethnic drugs on post-stroke mitochondrial MMP and Ca^2+^ or other ion levels (Kislin et al., 2017; Wilson et al., 2018; Zhang et al., 2019c). In conclusion, we were optimistic that abnormal opening of mitochondrial MPTP-induced apoptosis would become a potential target for stroke treatment by ethnic medicine. Further, we conceived and constructed the systematic process and program of drugs regulating mitochondrial MPTP to inhibit apoptosis in ischemic stroke, as shown in **Figure 5**. However, objectively speaking, no matter how many preclinical investigations were merely paving the way for screening mitochondrial MPTP targeted candidates, clinical trials with large samples and multi-center joint evaluation of the clinical efficacy of candidates were still necessary to be carried out.

## Author Contributions

XW conceived the study. YL, JS, RW, JB, YH, YZe, XM, and YZh reviewed and summarized the literatures. XW wrote the manuscript and drew all the figures. XW, ZW, and XM supervised the study and gave final approval of the version to be published. The final version of the manuscript was read and approved by all authors.

## Conflict of Interest

The authors declare that the research was conducted in the absence of any commercial or financial relationships that could be construed as a potential conflict of interest.

## Abbreviations

AIF, apoptosis-inducing factor; Apaf-1, apoptotic protease activating factor 1; ATF6, activating transcription factor-6; Bcl-2, B-cell lymphoma 2; BDNF, brain-derived neurotrophic factor; BrdU, 5-bromo-20-deoxyuridine; CHOP, C/EBP homologous protein; c-IAP1, cellular inhibitor of apoptosis 1; CREB, cyclic AMP response element binding protein; EndoG, endonuclease G; ERK, extracellular signal-regulated protein kinases; GFAP, glial fibrillary acidic protein; GluR1, glutamate receptor 1; GRP78, glucose regulator protein 78; HBMECs, human brain microvascular endothelial cells; HIF-1α, hypoxia-inducible factor-1 alpha; IκBα, nuclear factor of kappa light polypeptide gene enhancer in B-cells inhibitor, alpha; ICAM-1, intercellular cell adhesion molecule-1; IDH2, isocitrate dehydrogenase 2; IRE1, inositol-requiring protein 1; Keap1, Kelch-like ECH-associated protein 1; MAP-2, microtubule-associated protein-2; MCAO, middle cerebral artery occlusion; Mfn2, mitofusin-2; MMP-2, matrix metalloproteinase-2; MPTP, mitochondrial permeability transition pore; NeuN, neuron-specific nuclear; NMDAR1, N-methyl-D-aspartate receptor 1; NQO1, NAD(P)H, quinone oxidoreductase-1; Nrf2, nuclear factor erythroid-2-related factor 2; OCRs, oxygen consumption rates; OGD/R, oxygen glucose deprivation/reperfusion; OMM, outer mitochondrial membrane; PAF, platelet activating factor; PARP, poly ADP-ribose polymerase; PCN, primary cortical neurons; p-eIF2α, phospho-eIF2α; PERK, protein kinase RNA-like ER kinase; p-GSK-3β, phospho-glycogen synthase kinase-3β; PHN, primary hippocampal neurons; Pim-1, proto-oncogene serine/threonine-protein kinase; PKA, protein kinase A; p-PDK1, phosphoinositol-dependent kinase-1; PTEN, phosphatase and tensin homolog deleted on chromosome 10; Puma, p53 up-regulated modulator of apoptosis; SIRT3, silent mating type information regulation 2 homolog 3; STAT3, signal transducer and activator of transcription 3; TBARS, thiobarbituric acid reactive substances; TGF-β1, transforming growth factor; β-III-tubulin, microtubule element of the tubulin family; XBP-1, X-box-binding protein-1; ZO-1, zonula occludens-1.
