# Supplementary material for: Mitochondrial MPTP: A Novel Target of Ethnomedicine for Stroke Treatment by Apoptosis Inhibition
Source: Front Pharmacol. 2020 Mar 25;11:352. doi: 10.3389/fphar.2020.00352 (PMC7109312; doi:10.3389/fphar.2020.00352)
Supplement: Supplementary file 1 [file Table_1.docx]

**Supplementary TABLE 1 Detailed information of authorized products that may regulate MPTP to inhibit neuronal apoptosis in the treatment of ischemic stroke.**

| **NO.** | **Preparation Name** | **Main Compositions** | **Function** | **Usage and dosage** |
| --- | --- | --- | --- | --- |
| **1** | XueShuanTong injection | *Panax notoginseng* (Burk.) F. H. Chen | activating blood to remove stasis | i.v., 2-5 ml, diluted with 20~40 ml sodium chloride injection, q.d. or b.i.d.;  i.v. gtt., 2-5 ml, diluted with 250-500 ml 10% glucose injection, q.d. or b.i.d.;  i.m., 2-5 ml, q.d. or b.i.d. |
| **2** | Cerebralcare granule | *Angelica sinensis* (Oliv.) Diels, *Ligusticum chuanxiong* Hort., *Paeonia lactiflora* Pall., *Uncaria sinensis* (Oliv.) Havil., *Spatholobus suberectus* Dunn, *Prunella vulgaris* L., *Hyriopsis cumingii* (Lea), *Rehmannia glutinosa* Libosch., *Cassia tora* L., *Corydalis yanhusuo* W.T.Wang, *Asarum sieboldii* Miquel. | nourishing blood to calm liver, promoting blood circulation to remove meridian obstruction | p.o., 4 g once, t.i.d. |
| **3** | DangGui Jakyak san | *Atractylodes macrocephala* Koidz., *Alisma orientale* (Sam.) Juzep., *Poria cocos* (Schw.) Wolf, *Paeonia lactiflora* Pall., *Angelica sinensis* (Oliv.) Diels, *Ligusticum chuanxiong* Hort. | soothing liver to strengthen the spleen and relieve dampness, activating blood to remove stasis | p.o., 6 g once, t.i.d. |
| **4** | QianCao NaoMaiTong mixture | *Boschniakia himalaica*, *Lysimachia barystachys* | activating blood to remove stasis, eliminating phlegm by removing meridian obstruction | p.o., 10 ml once, t.i.d. |
| **5** | YiQi FuMai powder injection | *Panax ginseng* C. A. Mey, *Ophiopogon japonicus* (L. f) Ker-GawL., *Schisandra chinensis* (Turcz.) Baill. | supplement Qi and restore pulse, nourishing Yin to generate body fluid | i.v. gtt., q.d., 8 vials once, diluted with 250-500 ml 5% glucose or 0.9% sodium chloride injection. |
| **6** | TongXinLuo capsules | *Hirudo nipponica* Whitman, *Cryptotympana pustulata* Fabricius, *Steleophaga plancyi* (Boleny), *Buthus martensii* Karsch, *Scolopendra subspinipes mutilans* L. Koch, *Boswellia carterii* Birdw., *Dalbergia odorifera* T.Chen, *Borneolum syntheticum*, *Panax ginseng* C.A. Mey, *Paeonia lactiflora* Pall., *Ziziphus jujuba* Mill.Var.spinosa (Bunge) Hu H.F.Chou, *Santalum album* L. | benefiting Qi for activating blood circulation, activating meridians to stop pain | p.o., 2-4 granules once, t.i.d. |
| **7** | XingNaoJing injection | *Curcuma longa* L., *Moschus berezovskii* Flerov, Borneolum syntheticum, *Gardenia jasminoides* J. Ellis | heat-clearing and detoxicating, cooling blood fot promoting blood flow, activiting brain and regaining consciousness | i.m., 2-4 ml once, q.d. or b.i.d.; i.v. gtt., 10-20 ml once, diluted with 250-500 ml 5%-10% glucose or sodium chloride injection. |
| **8** | Pien Tze Huang capsules | *Panax notoginseng* (Burk.) F. H. Chen, *Bos taurus domesticus* Gmelin., *Moschus berezovskii* Flerov | heat-clearing and detoxicating, relieving pain and inflammation, activating blood to remove stasis | p.o., 2 granules once, 1 granule once for children aged one to five, t.i.d. |
| **9** | AnGong NiuHuang wan | *Bos taurus domesticus* Gmelin, *powder of Cornu bubali*, *Moschus berezovskii* Flerov, *Pteria martensii* (Dunker), Cinnabaris, Realgar, *Coptis chinensis* Franch., *Scutellaria baicalensis* Georgi, *Gardenia jasminoides* J. Ellis, *Curcuma Longa* L., *Borneolum synthcticum* | heat-clearing and detoxicating, relieving convulsion and inducing resuscitation | p.o., 1 pill once, q.d.; children under 3 years old a 1/4 pill, 4 to 6 years old a 1/2 pill, q.d. or following the doctor's advice. |
| **10** | XueSaiTong injection | *Panax notoginseng* (Burk.) F. H. Chen | activating blood to remove stasis, regulating meridians and collaterals | p.o., 2 granules once, b.i.d. |
| **11** | ZhenLong XingNao capsules | *Pteria martensii* (Dunker), *Bambusa textilis* McClure, *Crocus sativus* L., *Eugewia caryophyllata* Thunb., *Myristica fragrans* Houtt., *Amomurn kravanh* Pierre ex Gagnep*.*, *Amomum tsao-ko* Crevost et Lemaire, *Santalum album* L., *Pterocarpus santalinus, Aquilaria sinensis* ( Lour.) Gilg, *Terminalia chebula* Retz., *Terminalia bellirica* (Gaertn) Roxb., *Phyllanthus emblica* L., *Aucklandia lappa* Decne., *Cinnamomum cassia* Presl, *Piper longum* L., crab, Miace lapis aureus, *Cuminum cyminum* L., Bovls calculus artifactus, *Moschus* berezovskii Flerov, *Choerospondias axillaris* (Roxb.) Burtt et Hill, *Rhododendron anthopogonoides* Maxim., *Corydalis impatiens* (Pall.) Fisch, *Lagotis brachystachya Maxim.,* Fe, *Malva erticillata* L., *Glycyrrhiza uralensis* Fisch., *Nigella glandulifera* Freyn et Sint | activiting brain and regaining consciousness, heat-clearing and dredging collaterals | p.o., 2 granules once, q.d. or b.i.d. |
| **12** | ErShiWei ChenXiang pill | *Aquilaria sinensis* (Lour.) Gilg, *Eugewia caryophyllata* Thunb., Chaenomeles speciose (Sweet) Nakai, *Myristica fra grans* Houtt., *Carthamus tinctorius* L., *Choerospondias axillaris* (Roxb.) Burtt et Hill, *Inula recemosa* Hook. f., *Travertine, Cervus elaphus* Linnaeus, *Boswellia carterii* Birdw., *Hyriopsis cumingii* (Lea), *Aucklandia lappa* Decne., *Strychnos nux-vomica* L., *Terminalia chebula* Retz., *Lagotis brachystachya* Maxim., *Gossampinus malabarica* (DC.) Merr., *Phyllanthus emblica* L., *Dalbergia odorifera* T. Chen, *Lepus oiostolus* Hodgson, Bovls calculus artifactus | regulating and harmonizing Qi and blood, mind-tranquilizing | p.o., 3-4 g once, b.i.d. |
| **13** | DanHong injection | *Salvia miltiorrhiza* Bge., *Crocus sativus* L. | activating blood to remove stasis, regulating meridians and collaterals | i.m., 2-4 ml once, q.d. or b.i.d.; i.v., 4 ml once, diluted with 20 ml 50% glucose injection, q.d. or b.i.d.;  i.v. gtt., 20-40 ml once, diluted with 100-500 ml 5% glucose injection. |
| **14** | QingKaiLing injection | *Isatis indigotica* Fort., *Lonicera japonica* Thunb., *Hyriopsis cumingii* (Lea), *Scutellaria baicalensis* Georgi, *Gardenia jasminoides* J. Ellis, *cholic acid, hyodeoxycholic acid, Bubalus bubalis* Linnaeus | heat-clearing and detoxicating, eliminating phlegm by removing meridian obstruction, activiting brain and regaining consciousness | i.m., 2-4 ml a day; i.v. gtt., 20-40 ml a day for patients with severe cases, diluted with 200 ml 10% glucose injection or 100 ml sodium chloride injection. |
| **15** | NaoLuoTong capsules | *Salvia miltiorrhiza* Bge., *Ligusticum chuanxiong* Hort., *Astragalus membranaceus* (Fisch.) Bge. var. mongholicus (Bge.) Hsiao, Methyl hesperidin, Vitamin B6, Tolperisone Hydrochloride | benefiting Qi for activating blood circulation, regulating meridians and collaterals | p.o., 1-2 granules once, t.i.d. |
| **16** | AnNao tablet | *Bos taurus domesticus* Gmelin, *Sus scrofadomestica* Brisson., Cinnabars, *Cfnnamomwm camphora* (L.) Presl, *TaraxacMm mongolicum* Hand.-Mazz., *Pteria martensii* (Dunker), *Scutellaria baicalensis* Georgi, *Coptis chinensis* Franch., *Ganiema jasminoides* Ellis, Realgar, *Curcuma zvenyujin* Y. H. Chen et C. Ling, *Gypsum fibrosum,* Hematite, *Hyriopsis cumingii* (Lea), *Mentha haplocalyx* Briq. | heat-clearing and detoxicating, bringing solace and steadying the mind, regaining consciousness by eliminating phlegm, subduing the endogenous wind and sedatives | p.o., 4 tablets once, b.i.d. or t.i.d. |
